# SARS-CoV-2 transmission with and without mask wearing or air cleaners in schools in Switzerland: A modeling study of epidemiological, environmental, and molecular data

**DOI:** 10.1371/journal.pmed.1004226

**Published:** 2023-05-18

**Authors:** Nicolas Banholzer, Kathrin Zürcher, Philipp Jent, Pascal Bittel, Lavinia Furrer, Matthias Egger, Tina Hascher, Lukas Fenner

**Affiliations:** 1 Institute of Social and Preventive Medicine, University of Bern, Bern, Switzerland; 2 Department of Infectious Diseases, Inselspital, Bern University Hospital, University of Bern, Bern, Switzerland; 3 Institute for Infectious Diseases, University of Bern, Bern, Switzerland; 4 Centre for Infectious Disease Epidemiology and Research, Faculty of Health Sciences, University of Cape Town, Cape Town, South Africa; 5 Population Health Sciences, Bristol Medical School, University of Bristol, Bristol, United Kingdom; 6 Institute of Educational Science, University of Bern, Bern, Switzerland; Edinburgh University, UNITED KINGDOM

## Abstract

**Background:**

Growing evidence suggests an important contribution of airborne transmission to the overall spread of Severe Acute Respiratory Syndrome Coronavirus 2 (SARS-CoV-2), in particular via smaller particles called aerosols. However, the contribution of school children to SARS-CoV-2 transmission remains uncertain. The aim of this study was to assess transmission of airborne respiratory infections and the association with infection control measures in schools using a multiple-measurement approach.

**Methods and findings:**

We collected epidemiological (cases of Coronavirus Disease 2019 (COVID-19)), environmental (CO_2_, aerosol and particle concentrations), and molecular data (bioaerosol and saliva samples) over 7 weeks from January to March 2022 (Omicron wave) in 2 secondary schools (*n* = 90, average 18 students/classroom) in Switzerland. We analyzed changes in environmental and molecular characteristics between different study conditions (no intervention, mask wearing, air cleaners). Analyses of environmental changes were adjusted for different ventilation, the number of students in class, school and weekday effects. We modeled disease transmission using a semi-mechanistic Bayesian hierarchical model, adjusting for absent students and community transmission.

Molecular analysis of saliva (21/262 positive) and airborne samples (10/130) detected SARS-CoV-2 throughout the study (weekly average viral concentration 0.6 copies/L) and occasionally other respiratory viruses. Overall daily average CO_2_ levels were 1,064 *±* 232 ppm (*±* standard deviation). Daily average aerosol number concentrations without interventions were 177 *±* 109 1/cm^3^ and decreased by 69% (95% CrI 42% to 86%) with mask mandates and 39% (95% CrI 4% to 69%) with air cleaners. Compared to no intervention, the transmission risk was lower with mask mandates (adjusted odds ratio 0.19, 95% CrI 0.09 to 0.38) and comparable with air cleaners (1.00, 95% CrI 0.15 to 6.51).

Study limitations include possible confounding by period as the number of susceptible students declined over time. Furthermore, airborne detection of pathogens document exposure but not necessarily transmission.

**Conclusions:**

Molecular detection of airborne and human SARS-CoV-2 indicated sustained transmission in schools. Mask mandates were associated with greater reductions in aerosol concentrations than air cleaners and with lower transmission. Our multiple-measurement approach could be used to continuously monitor transmission risk of respiratory infections and the effectiveness of infection control measures in schools and other congregate settings.

## Abstract 2

Nicolas Banholzer and team use a variety of data to investigate the transmission of SARS-CoV-2 in two secondary schools in Switzerland in the presence and absence of mask wearing and air cleaners.

## Author summary

**Why was this study done?:**

- Public health authorities worldwide closed businesses and schools during the Coronavirus Disease 2019 (COVID-19) pandemic.
- The closure of schools has been most intensely debated.
- The contribution of school children to Severe Acute Respiratory Syndrome Coronavirus 2 (SARS-CoV-2) transmission and the role of school rooms remain unknown.

**What did the researchers do and find?:**

- We used molecular, environmental, and epidemiological data to understand the transmission of the virus causing COVID-19 (SARS-CoV-2) in 2 secondary schools (90 students) in Switzerland in the presence and absence of mask wearing and air cleaners.
- We detected SARS-CoV-2 in aerosols in the air and saliva samples from the students throughout the study.
- Aerosol and particle concentrations were on average 70% lower with mask mandates and 40% lower with air cleaners.
- The transmission model estimated that between 2 and 19 infections could be avoided during the study period with mask wearing.

**What do these findings mean?:**

- Molecular analyses indicated sustained airborne SARS-CoV-2 transmission.
- Mask wearing may be more effective than air cleaners in reducing aerosol concentrations and transmission of SARS-CoV-2.
- This approach can be used to assess transmission dynamics and the effectiveness of infection control measures in reducing transmission of respiratory infections during future epidemics.

## 1 Introduction

The spread of respiratory infections such as Severe Acute Respiratory Syndrome Coronavirus 2 (SARS-CoV-2) and influenza is difficult to control [1]. Person-to-person transmission mainly occurs through exhaled respiratory particles containing the respective pathogen, particularly via aerosols (defined as respiratory particles <100 μm [2,3]), rather than through larger droplets >100 μm. Multiple reports have provided evidence that a considerable part of SARS-CoV-2 transmission is likely to happen through small respiratory particles (<5 μm, also called fine aerosols), allowing for longer suspension times and airborne transmission at short (1 to 2 m) and long ranges (>2 m) [4–6]. Growing evidence suggests they contribute importantly to the overall spread of SARS-CoV-2 in indoor congregate settings such as clinics, workplaces, and schools [3,6–8].

Public authorities worldwide closed businesses and schools during the Coronavirus Disease 2019 (COVID-19) pandemic [9,10], but the closure of schools was particularly contentious. While the negative impact of school closures on student well-being and mental health is well documented [11], the role of children and adolescents in transmitting SARS-CoV-2 is less clear [12]. A study in Germany estimated that school contacts contributed between 2% and 20% to the overall transmission of SARS-CoV-2 in the population [13]. Studies on the effectiveness of government interventions at the population level are inconclusive regarding the effects of school closures in different epidemic waves [10,14]. The introduction of compulsory face mask wearing [13,15] and improved ventilation [15] in schools was associated with a lower incidence of COVID-19. In addition, the installation of portable HEPA-air filtration devices (air cleaners) was shown to remove SARS-CoV-2 bioaerosols in a hospital ward [16]. Finally, a simulation study of exhaled SARS-CoV-2 bioaerosols in an indoor space demonstrated the efficacy of mask wearing and portable air cleaners in reducing aerosols [17].

We used a multiple-measurement approach to study the transmission of SARS-CoV-2 and other respiratory viruses in school rooms. We hypothesized that our approach could demonstrate transmission (indicated by exposure to bioaerosols and epidemiological data) and that infection control measures (mask wearing and air cleaners) would reduce the concentration of aerosols and respiratory particles and lower the risk of infection for students in school rooms. We collected epidemiological (cases of respiratory diseases), environmental (CO_2_, aerosol, and particle concentrations), and molecular data (detection of respiratory viruses in bioaerosol and human saliva samples) over a seven-week study period from January to March 2022 in 2 secondary schools in Switzerland. We analyzed changes in environmental and molecular characteristics and estimated the probability of infection with SARS-CoV-2 during 3 different study conditions with and without infection control measures (general mask wearing and air cleaners).

## 2 Methods

### 2.1 Study setting and design

**Fig 1:**
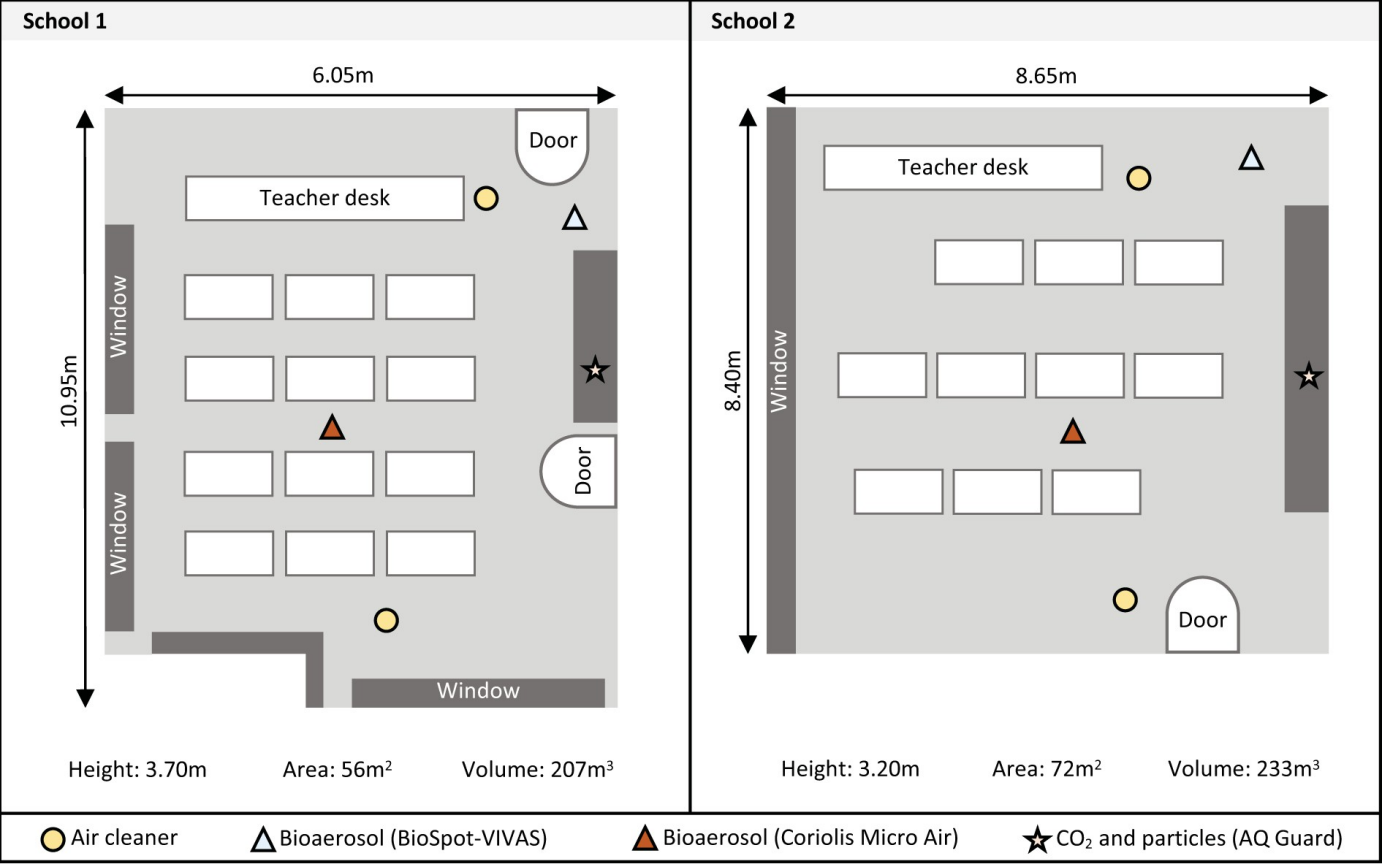
Study setting. Schematic study setup of classrooms where environmental data was collected in each school. One air cleaner was placed in the front and the other in the back of classrooms. All devices were placed at the head level of students when they were seated. Both classrooms were not equipped with an active HVAC (heating, ventilation, air conditioning) system, but were ventilated using passive window ventilation. For School 1, ventilation was additionally assisted by a CO_2_ guided opener of a small window at the top.

We collected data in 2 secondary schools (age of students 13 to 15 years) over a seven-week study period from January 24 to March 26 (School 1) and March 18 (School 2), 2022. Both schools are located in the Canton of Solothurn, Switzerland, and have 1,500 (School 1) and 700 (School 2) students. Epidemiological data were collected in the 5 classes (School 1: classes A/B, C; School 2: classes D, E), and environmental and molecular data were collected in 2 classrooms (School 1: A/B, School 2: D). In School 1, the same classroom was used by 2 classes A/B due to half-class teaching. Fig 1 shows the schematic study setup. This study is reported as per the Strengthening the Reporting of Observational Studies in Epidemiology (STROBE) guideline (S1 Checklist).

### 2.2 Study interventions

**Fig 2:**
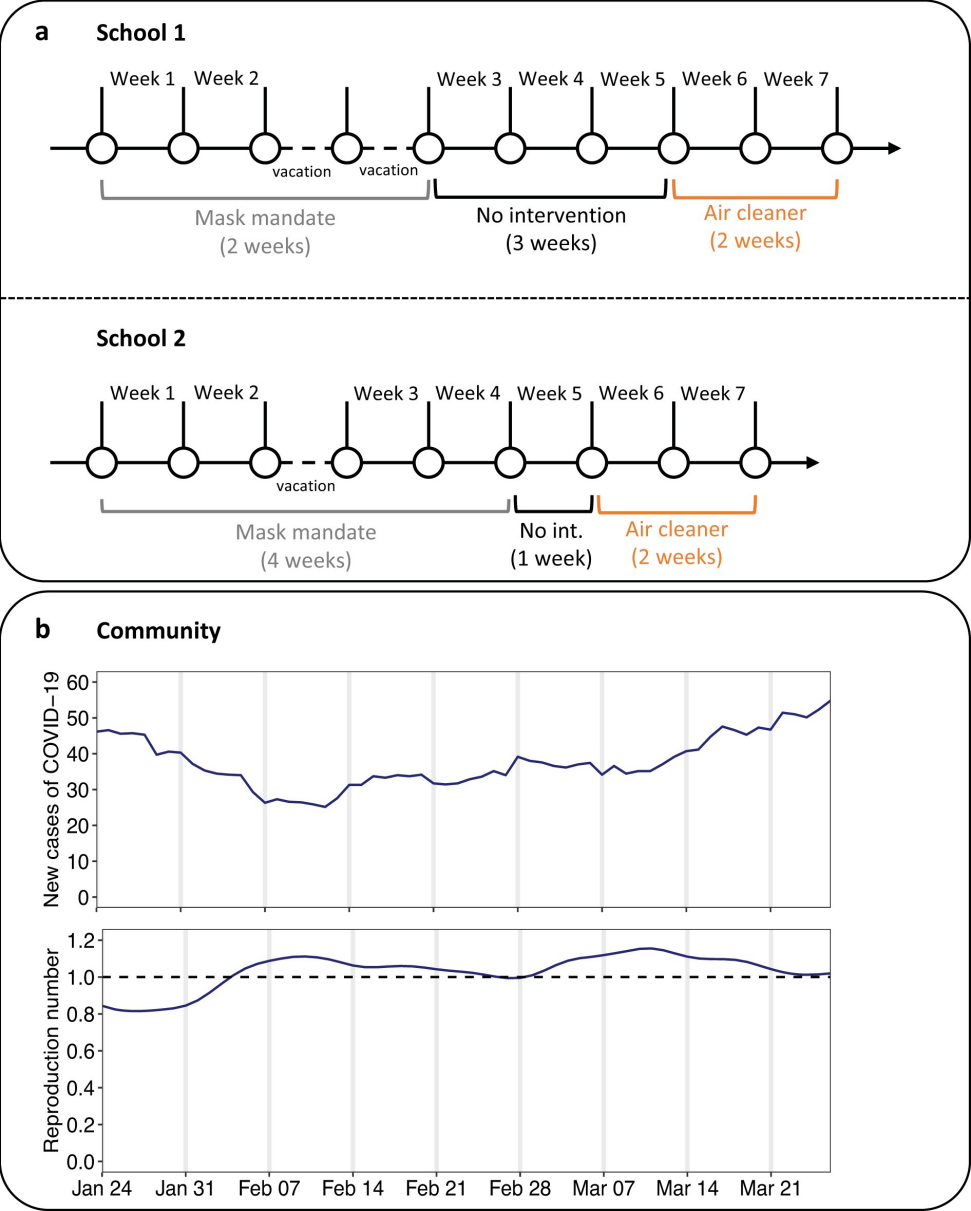
Study design and community transmission during the study period. **(a)** Study conditions over the seven-week study period. **(b)** Number of new cases of COVID-19 across all age groups (7-day moving average) and the reproduction number (average of the median published estimate) in the Canton of Solothurn [18] over the study period.

We distinguished 3 conditions (Fig 2): (i) wearing face masks as mandated by the public health authorities at that time (*Mask mandate*; typically Type II and Type IIR masks, although community masks were also allowed); (ii) standard condition following the lifting of mask mandates (*No intervention*); and (iii) environmental intervention using commercially available portable HEPA- filtration devices (*Air cleaner*; Xiaomi Mi Air Pro 70m2, Shenzhen, China; approx. USD 250 per device, running at 2 *×* 600 m^3^/h clean air delivery rate). Mask mandates applied to all classes (including teachers) and were generally well adhered to. In School 2, mask wearing continued for 1 week after the mandate was lifted (week 4). Air cleaners were only installed in 2 classrooms with bioaerosol and environmental sampling. Passive window ventilation occurred per recommendations of the national public health authorities during all study conditions.

### 2.3 Data collection

#### Epidemiological data

At baseline, we collected aggregated data on age, sex, and COVID-19 vaccination status in the participating classes. We inferred data on the number of suspected and confirmed cases of COVID-19 based on the reported number of students absent from school due to sickness probably related to COVID-19 (Text C and Tables A–E in S1 Appendix). Reports about absences were entered electronically into a REDCap database [19,20]. Both schools participated in repetitive weekly testing for SARS-CoV-2. The saliva samples were transported to the laboratory and stored at *−*80°C until further processed [21–23].

#### Environmental data

*CO_2_ and particle measurements.* An air quality device (AQ Guard, Palas GmbH, Karlsruhe, Germany) continuously measured indoor CO_2_ levels, aerosol number concentrations (particle diameter between 175 nm to 20 μm) and particle mass concentrations (PM in μgm^*−*3^; PM_1_, PM_2.5_, PM_4_, PM_10_, i.e., particles of sizes <1 to <10 μm). This device has been used in previous work [24,25]. Data were filtered according to the times students were in the classroom, which were monitored together with the time that windows were opened.

*Bioaerosol sampling.* We collected airborne respiratory viruses in the classroom with a bioaerosol sampling device (BioSpot-VIVAS, Aerosol Devices, Ft. Collins, Colorado, United States of America). This device samples airborne virus particles into a viral transport medium (VTM) using a laminar flow water-based condensation method. In parallel, we also used the Coriolis Micro Air (Bertin Instruments Montigny-le-Bretonneux, France) sampler, running at 200 l/min and collecting into 15 mL PBS as previously reported in clinic settings [26]. BioSpot-VIVAS operated throughout lessons while Coriolis Micro Air only operated shortly before and during break times to reduce noise exposure (approximately 60 min/day). The removable parts of both sampling devices were regularly autoclaved to avoid contamination. At the end of the day, samples were transported to the Institute for Infectious Diseases (IFIK) and stored at *−*80°C. At the end of the study period, the Xiaomi HEPA filters were carefully removed and 20 swabs were taken from each filter and stored at *−*80°C until further processed.

### 2.4 Laboratory and molecular analyses

Prior to the real-time (RT)-PCR analysis, daily bioaerosol samples were combined for each sampling device and filtered using Amicon Ultra-15 Centrifugal Filters with Ultracel 10,000 Dalton molecular weight cutoffs filters (UFC9010; MilliporeSigma, Burlington, USA) to a volume of 1 mL. The human saliva samples were directly analyzed without prior filtration. The Allplex RV Master Assay (Seegene, Seoul, South Korea) was used to detect a panel of 19 respiratory viruses (Text A in S1 Appendix), including SARS-CoV-2. Viral genome load (VGL) of specimens was quantified using standardized dilution series and reported in genomic equivalent copies/L. For positive samples, we performed targeted sequencing of viral RNA to compare genetic relatedness between SARS-CoV-2 strains detected in the air and human samples [27]. However, we were unable to amplify and sequence any of the gene targets in the bioaerosol samples due to low RNA concentrations.

### 2.5 Statistical analyses and modeling

#### Number of new cases of COVID-19 by date of symptom onset

The daily number of new cases of COVID-19 was inferred based on the number of students absent from school. Confirmed cases referred to absences due to a positive laboratory test result or isolation. Suspected cases referred to absences due to sickness probably related to COVID-19. Absences due to other known reasons were excluded. We used a probabilistic simulation approach (Text D in S1 Appendix) to estimate the number of suspected cases that were cases of COVID-19 and the date of symptom onset, which was not always reported by students. We generated 100 datasets for the daily number of new cases of COVID-19. Subsequent analyses were performed on each of these datasets and we report the mean of estimation results if not stated otherwise. Cases in teachers were excluded as teachers taught multiple classes and had different exposure.

#### Aerosol and particle concentrations

We computed the average concentrations per day and compared these between study conditions. We report the mean *±* standard deviation. We further estimated the reduction in concentrations using Bayesian log-linear regression models (Text H in S1 Appendix), adjusting for the ventilation rate (computed from indoor CO_2_ levels; see Text I in S1 Appendix), the daily number of students in class, school, and weekday effects.

#### Risk of transmission

**Fig 3:**
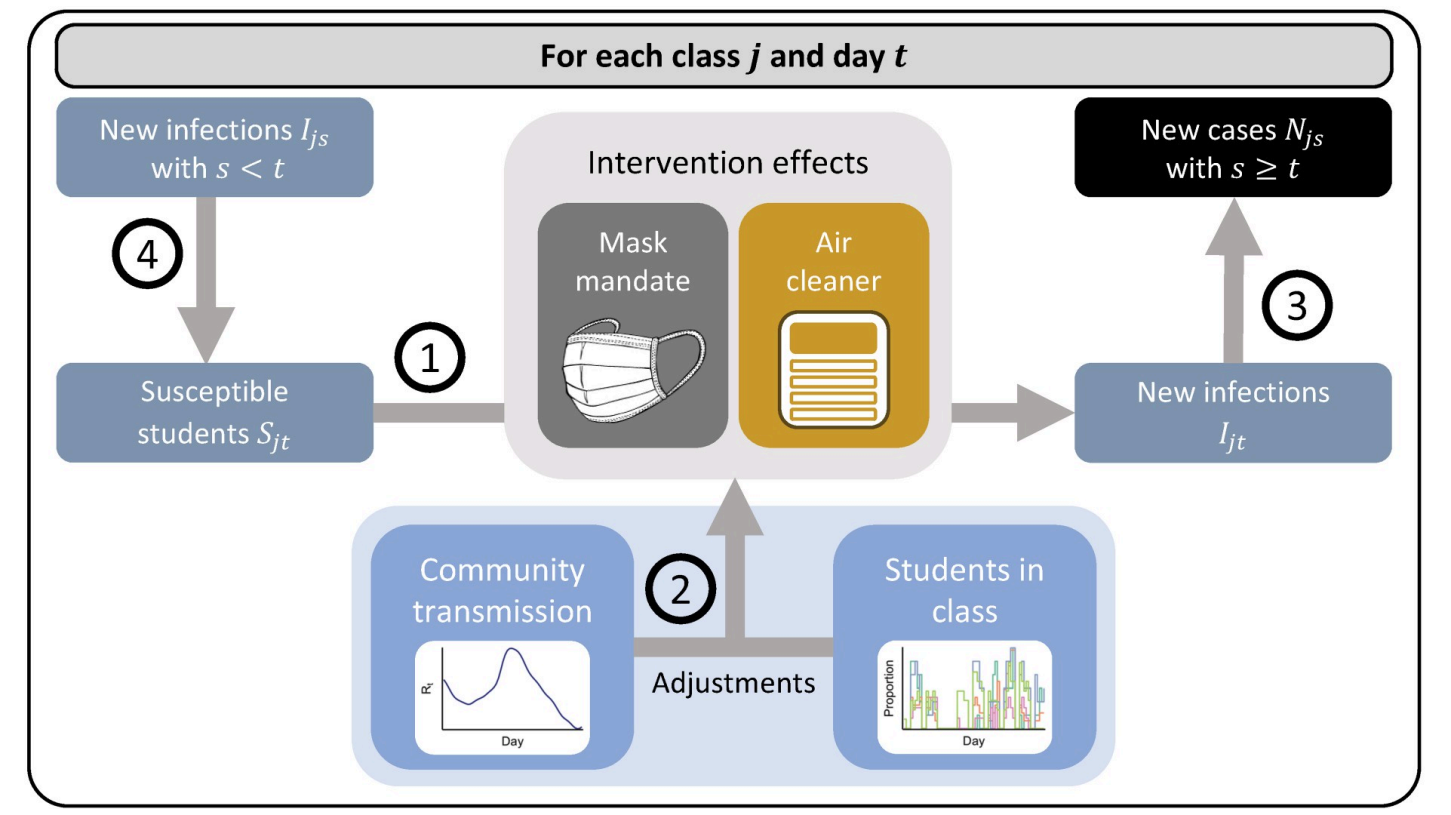
Visual summary of the structure of the Bayesian semi-mechanistic hierarchical model. (1) The number of new infections was modeled as a function of the number of susceptible students, the class-specific daily rate of infections, and the reductions from active infection control measures; (2) the effects of control measures were adjusted for transmission in the community and the proportion of students in class; (3) the observed number of new cases was computed as the weighted sum of the number of new infections in the previous days; and (4) the number of susceptible students was computed as the total number of students minus the cumulative sum of infections in the previous days.

We estimated daily transmission of SARS-CoV-2 with a Bayesian semi-mechanistic hierarchical model [9] (Fig 3): (i) We modeled the number of new infections as a function of susceptible students in each class and day, where the probability of infection can vary by study condition. (ii) We adjusted the estimated effects of interventions for the daily proportion of all absent students and the effective reproduction number in the community. (iii) The number of new cases was computed as the weighted sum of the number of new infections in the previous days. (iv) The number of susceptible students was computed by removing the number of students who have already been infected.

A detailed description of the transmission model and choice of priors for all model parameters are provided in Text E in S1 Appendix. Model parameters were estimated with a Bayesian approach (Text F in S1 Appendix). Specifically, Markov chain Monte Carlo (MCMC) sampling was used as implemented by the Hamiltonian Monte Carlo algorithm with the No-U-Turn Sampler (NUTS) [28]. If not stated otherwise, we report posterior means and credible intervals (CrIs) based on the 50%, 80%, and 95% quantiles of the posterior samples, respectively. We estimated the total number of avoided infections for each intervention by computing the difference between the estimated number of infections in the presence and absence of interventions (counterfactual scenario).

#### Software

All analyses were performed in R software (version 4.2.0) [29] and modeling in Stan (version 2.21.0) [30]. The code is available from https://github.com/nbanho/mcid.

### 2.6 Ethics statement

The Ethics Committee of the Canton of Bern, Switzerland, approved the study (reference no. 2021–02377). For the saliva samples, we included all students who were willing to participate and obtained written informed consent from their caregivers.

## 3 Results

The study population consisted of 90 students (39 female, 51 male; Table 1). Of these, 27 students were fully vaccinated and 34 students had recovered from an infection within the last year. Over the seven-week study period (3,150 student-days in total), students were absent from school for 644 days (20% of total) of which 147 days (23% of absences) were due to isolation related to COVID-19, and 247 (38% of absences) were due to sickness. Overall, there were 35 confirmed and 73 suspected cases of COVID-19, exceeding the number of students in some classes (Table C in S1 Appendix). This suggests that only a proportion of suspected cases were actual cases of COVID-19 as it is unlikely that students were infected twice. Accordingly, we estimated the actual number of cases of COVID-19 across schools to be 55 (95% CrI 35 to 76).

**Table 1:** Overview of the study population, number of COVID-19 cases, and person-days of absences.

	School 1	School 2	Total
**Students**	**52 (58%)**	**38 (42%)**	**90 (100%)**
*Sex*			
Female	20 (38%)	19 (50%)	39 (43%)
Male	32 (62%)	19 (50%)	51 (57%)
*Vaccination status*			
Vaccinated	16 (31%)	11 (29%)	27 (30%)
Not vaccinated	36 (69%)	27 (71%)	63 (70%)
*Recovery status*			
Recovered last year	25 (48%)	9 (24%)	34 (38%)
Not recovered	27 (52%)	29 (76%)	56 (62%)
**Absent person-days**	**334 (52%)**	**310 (48%)**	**644 (100%)**
Isolation	109 (33%)	38 (12%)	147 (23%)
Sickness	95 (28%)	152 (49%)	247 (38%)
Quarantine	55 (17%)	5 (2%)	60 (9%)
Other	75 (22%)	115 (37%)	190 (30%)
**COVID-19 cases**	**78 (19%)**	**30 (13%)**	**108 (100%)**
Confirmed	25 (32%)	10 (33%)	35 (32%)
Suspected	53 (68%)	20 (67%)	73 (68%)

### 3.1 Molecular analyses

**Fig 4:**
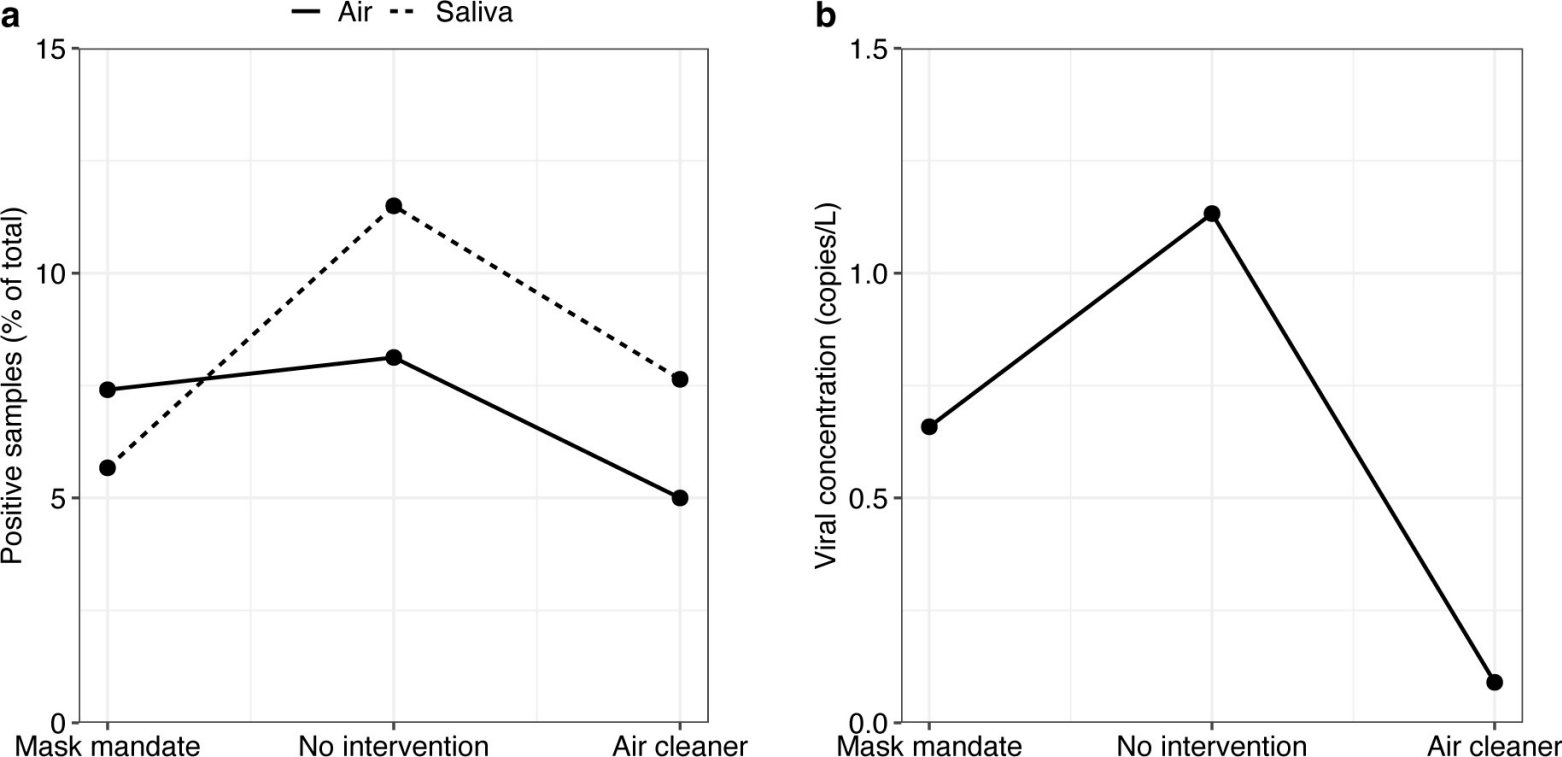
Analysis of molecular data and comparison by study condition. **(a)** Proportion of positive airborne (solid line) and saliva (dashed line) samples (average per week). **(b)** Viral concentration in positive airborne samples from BioSpot-VIVAS (average per week).

We analyzed 262 saliva, 130 bioaerosol samples and swabs from the filters of air cleaners (20 swabs per filter) in 2 classrooms. Overall, there were 21 positive saliva and 10 positive airborne samples. We detected SARS-CoV-2, adenovirus, and influenza virus (Table 2 and Tables A-B in S1 Appendix). SARS-CoV-2 made up the vast majority of positive saliva (19 out of 21) and bioaerosol samples (9 out of 10). We found 4 positive air-saliva samples in the same respective week (3 SARS-CoV-2 and 1 adenovirus), suggesting they were paired samples. We also detected SARS-CoV-2 and influenza viruses from the HEPA filters of the air cleaners. The number of positive saliva and airborne SARS-CoV-2 samples per week varied by study condition (Fig 4A). Without interventions, the proportion of positive samples per week was 11.5% for saliva and 8.1% for airborne samples. These proportions were lower with mask mandates (saliva: 5.7%, air: 7.1%) and air cleaners (saliva: 7.7%, air: 5.0%). The weekly average viral concentration of positive samples was 0.6 copies/L. There were also differences in viral concentration between study conditions (Fig 4B). Without interventions, it was 1.1 copies per liter per week, which was higher than with mask mandates (0.7 copies/L per week) and air cleaners (0.1 copies/L per week).

**Table 2:** Analysis of molecular data and the number of positive and negative saliva and airborne samples in each school.

	Saliva				Air				Air cleaner (HEPA filter†)			
	School 1		School 2		School 1		School 2		School 1		School 2	
**Total**	**173**		**89**		**68**		**62**					
Negative	160	(92%)	81	(91%)	64	(94%)	56	(90%)				
Positive	13	(8%)	8	(9%)	4	(6%)	6	(10%)				
**Positive**	**13**		**8**		**4**		**6**		**2**		**6**	
SARS-CoV-2	12	(92%)	7	(88%)	4	(100%)	5	(83%)	2	(100%)	4	(66%)
Influenza	1	(8%)	0	(0%)	0	(0%)	0	(0%)	0	(0%)	1	(17%)
Adeno	0	(0%)	1	(12%)	0	(0%)	1	(17%)	0	(0%)	1	(17%)

† A total of 20 swabs were taken from each filter at the end of the study period.

### 3.2 Analysis of aerosol and particle concentrations

**Fig 5:**
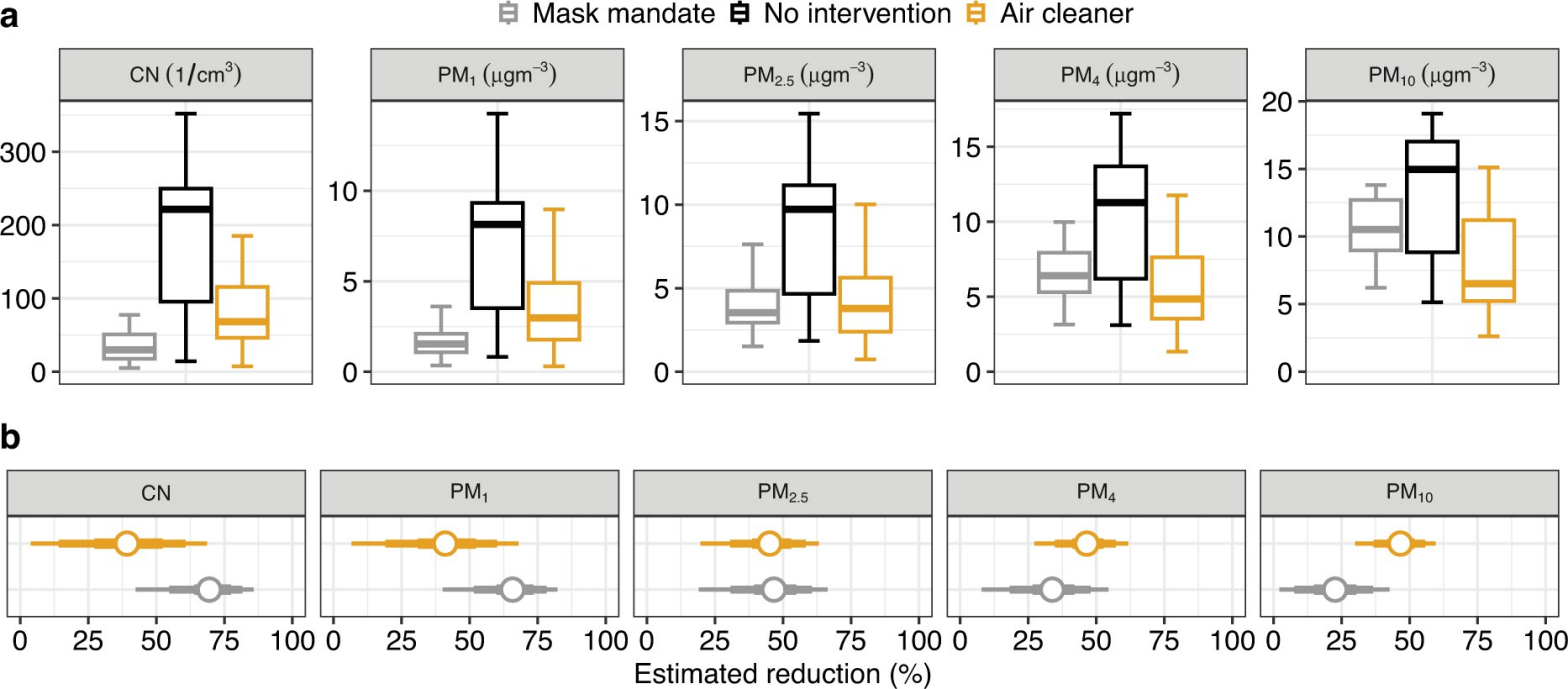
Analysis of particle concentrations and comparison by study condition. **(a)** Boxplot of the daily average values for aerosol number concentration (CN in 1/cm^3^) and particle mass concentration (PM for particles of sizes <1 to <10 μm, respectively in μgm^*−*3^). Results for CO_2_ and other environmental variables are provided in Text J in S1 Appendix. **(b)** Estimated reduction in aerosol number and particle mass concentrations with interventions (posterior mean as dot and 50%, 80%, and 95% CrI as lines, respectively).

Particle concentrations differed by study condition (Fig 5A). Aerosol number concentrations were lower with mask mandates (mean 49 *±* 52 1/cm^3^ standard deviation) and air cleaners (84 *±* 56 1/cm^3^) than without interventions (177 *±* 109 1/cm^3^). Similarly, particle mass concentrations (e.g., PM_1_) were lower with mask mandates (2.0 *±* 1.6 μgm^*−*3^) and air cleaners (3.8 *±* 2.9 μgm^*−*3^) than without interventions (6.9 *±* 4.1 μgm^*−*3^). Overall daily average CO_2_ levels were 1,064 *±* 232 ppm. CO_2_ levels without interventions (953 *±* 198 ppm) were slightly lower than with mask mandates (1,155 *±* 237 ppm) and air cleaners (1,088 *±* 224 ppm), indicating increased ventilation through outdoor air exchange (Fig I in S1 Appendix), although the differences in the daily proportion of time that windows were opened between no intervention and mask mandates (0.03, 95% CrI *−*0.07 to 0.12) and between no intervention and air cleaners (0.00, 95% CrI *−*0.11 to 0.11) were indistinguishable from zero. When adjusting for different ventilation rates, the number of students in class, school effects, and weekday effects, the aerosol number concentration decreased by 69% (95% CrI 42% to 86%) with mask mandates and by 39% (95% CrI 4% to 69%) with air cleaners (Fig 5B and Table H in S1 Appendix). The concentration of smaller particles (PM_1_ and PM_2.5_) was more reduced during mask mandates, and the concentration of larger particles (PM_4_ and PM_10_) was more reduced during air cleaners.

### 3.3 Estimating transmission risk of SARS-CoV-2

**Fig 6:**
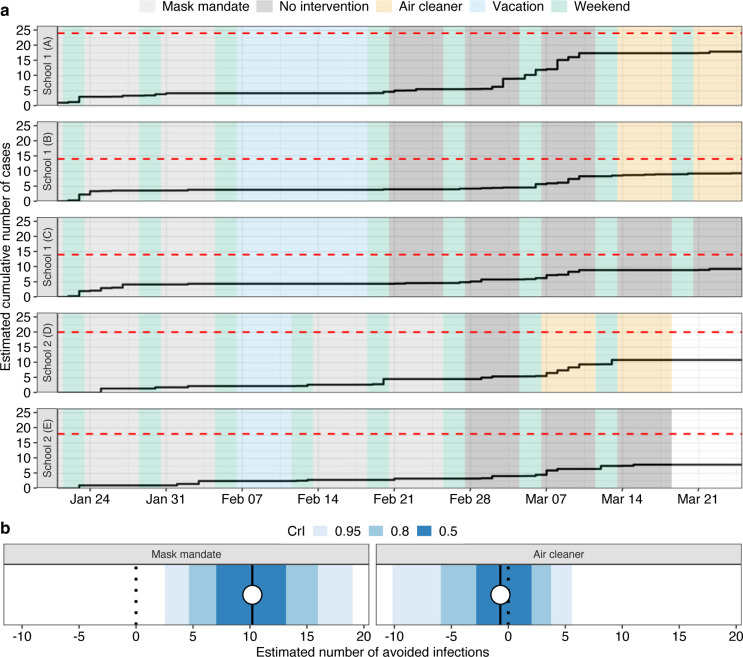
Analysis of epidemiological data and estimated transmission by study condition using the Bayesian hierarchical transmission model. **(a)** Estimated mean cumulative number of cases for each school class after probabilistic simulation accounting for uncertainty in suspected cases and the date of symptom onset (Text D in S1 Appendix). Dotted red lines indicate the number of students per class. A comparison of the estimated number of new cases after probabilistic simulation with the observed number of new confirmed and suspected cases is shown in Fig C in S1 Appendix. **(b)** Estimated number of avoided infections associated with interventions across schools (posterior mean as dot and 50%, 80%, and 95% CrI as shaded areas) based on the Bayesian hierarchical transmission model. Detailed estimation results are provided in Text G in S1 Appendix.

The cumulative number of cases of COVID-19 increased considerably in all classes without intervention and the large majority of students has been infected in School 1 by the time air cleaners were installed, except for class D in School 2 (Fig 6A). Based on our Bayesian transmission model, the probability of getting infected was lower with mask mandates than without interventions (adjusted odds ratio 0.19, 95% CrI 0.09 to 0.38), and comparable with air cleaners (1.00, 95% CrI 0.15 to 6.51). Excluding suspected cases from the model, these probabilities were similar for both mask mandates (0.21, 95% CrI 0.08 to 0.50) and air cleaners (0.98, 95% CrI 0.14 to 6.74), although with greater uncertainty. Considering both confirmed and suspected cases, mask mandates were associated with a considerable number of avoided infections (9.98, 95% CrI 2.16 to 19.00), but not air cleaners (Fig 6B). As an additional analysis, we used a modified Wells–Riley equation [31] and assumed that the change in the rate of emitted infectious quanta was proportional to the estimated reduction in the aerosol number concentration, while other parameters were kept constant under all study conditions (Text K in S1 Appendix). Accordingly, the daily risk of infection for a 6 h school day was 1.0% (95% CrI 0.4% to 1.9%) with mask mandates and 1.9% (95% CrI 1.0% to 3.0%) with air cleaners, compared to a 3.1% risk without interventions (Fig J in S1 Appendix).

## 4 Discussion

We collected epidemiological, environmental, and molecular data to estimate transmission of respiratory infections in schools and assessed the association with infection control measures. Airborne detection of SARS-CoV-2 documented sustained SARS-CoV-2 transmission. Bioaerosol SARS-CoV-2 concentrations with general mask mandates were lower, and SARS-CoV-2 was detected on the filters of air cleaners. Both interventions were associated with significantly lower aerosol number and particle mass concentrations. The Bayesian transmission model using epidemiological data estimated that mask mandates avoided SARS-CoV-2 infections, but not air cleaners.

Our study demonstrated airborne detection of SARS-CoV-2 in schools. Although sampling and molecular detection of infectious bioaerosols are challenging and there is no agreed standard [16,32,33], it provides important evidence on the airborne transmission of respiratory pathogens. So far, viral RNA in airborne samples of SARS-CoV-2 was mainly found in hospitals and healthcare facilities [34]. A related study in 2 South African schools detected airborne *Mycobacterium tuberculosis* [35]. Tuberculosis was the leading cause of death worldwide due to an infectious disease prior to the COVID-19 pandemic. An improved understanding of airborne transmission and the effectiveness of interventions can benefit the control of both infectious diseases [36]. Our study provides evidence on airborne SARS-CoV-2 viral transmission and the potential effects of interventions in schools based on airborne and saliva samples from students. In our study, positive samples mostly pertained to SARS-CoV-2, but we occasionally detected other respiratory viruses such as adenovirus and influenza. General public health measures during the study likely suppressed the spread of other respiratory viruses. It also must be noted that the detected viral concentrations were low, as shown by high cycle thresholds (CTs) in the RT-qPCR results. The molecular presence of other viral pathogens cannot be excluded. The low sensitivity for detection of airborne pathogens by molecular assays is a well-documented challenge [16,33].

An experimental study demonstrated the effectiveness of infection control measures (universal face mask wearing and air cleaners) using simulated exhaled SARS-CoV-2 bioaerosols in a closed indoor space [17]. In contrast, we studied infection control measures in a real-life setting and demonstrated their effectiveness using a multiple-measurement approach and obtain similar results. Our findings also align with existing evidence from population-level studies showing that the incidence of COVID-19 was lower in schools with mask use [13,15] and improved ventilation [15]. Similarly, a field study showed that adequate ventilation was associated with reduced incidence of tuberculosis, a strictly airborne disease, in a university building [37]. Altogether, these findings support arguments in favor of multiple intervention strategies to address airborne transmission of respiratory infections in crowded indoor settings [38].

Indoor ventilation is one of the key determinants of airborne transmission [2,3], but schools are often poorly ventilated [24,39]. We showed that the concentration of both larger and smaller particles were lower with air cleaners, in line with findings about their effectiveness in hospitals [16] and simulated indoor environments [17]. The detection of viruses on the filters of air cleaners further supports the evidence on the effective removal of bioaerosols. However, it was difficult to estimate changes in transmission following air cleaners because they were installed at the end of the study period when presumably a large proportion of students were already infected with SARS-CoV-2. Furthermore, air cleaners could not prevent transmission outside the classrooms (e.g., during breaks and outside classes), which limits their effectiveness compared to masks that had to be worn in all indoor settings following the general mask mandate. In addition, physicochemical properties of aerosols, environmental factors, and the distance to infectious people determine the survival of airborne particles and the loss of infectivity over time [3]. Thus, a predominance of close range high particle density aerosol transmission of SARS-CoV-2 in school settings could further explain why air cleaners were not associated with reduced transmission. Our study used portable, affordable air cleaners that could be implemented at scale, rather than large cleaners [25]. Noise exposure and a lack of acceptance by teachers [40] may still prevent their widespread use. Although not specifically assessed, we perceived good acceptance of our air cleaners during the short study period. Nevertheless, investments in professional building ventilation systems should be preferred to air cleaners in the long term [41].

School closures during the COVID-19 pandemic have been intensely debated as children and adolescents are particularly vulnerable to the negative impact of such interventions on their well-being and mental health [11]. Furthermore, numerous studies have examined the role of children in transmitting SARS-CoV-2 [12] and it remains unclear to what extent transmission of SARS-CoV-2 occurs in schools [42]. These findings contrast with studies of influenza viruses showing that school children may drive the seasonal influenza epidemic. Community studies in the US demonstrated that influenza transmission rates in children and adolescents were high in schools and that they easily transmit influenza viruses to household members and into their communities [43–45]. Our study suggests that also transmission of SARS-CoV-2 occurs to a considerable extent in schools.

Our study has several limitations. First, aerosol measurements and molecular detection of pathogens in bioaerosol samples document exposure, but not transmission and the direction of transmission (human to air, air to human). Nevertheless, paired saliva and airborne samples suggest that infected students exhaled infectious particles into the air, indicating a considerable transmission risk in the school rooms. Second, a comparison of viral concentration between study conditions should be interpreted with care due to the technical limitations of molecular detection in bioaerosol samples, and because the number of possibly infectious (and thus susceptible) students decreased towards the end of the study. Third, our epidemiological analysis is based on observational data, thus subject to potential confounding, e.g., the incidence of COVID-19 (cases per week) in the community varied over the study period. However, levels were high throughout and included 2 Omicron subvariant peaks. Community transmission was also considered in our Bayesian transmission model. CO_2_ levels were not considered in the model but the levels were slightly lower without interventions, suggesting that lower ventilation during intervention phases may have actually reduced the estimated effectiveness of mask mandates and air cleaners. Fourth, epidemiological data may not always be complete due to the underreporting of COVID-19 among absent students. We thus used a probabilistic approach to estimate the proportion of suspected cases being actual cases of COVID-19 and the date of symptom onset where it was not reported. While this allows us to consider uncertainty in the observed data, the estimated effects of interventions will be less precise as reflected in larger uncertainty intervals. Fifth, the ordering of study conditions was the same across classes, mainly because our study period coincided with the lifting of general mask mandates. The effectiveness of infection control measures may thus be affected by their timing. Future studies could vary the ordering of interventions in each class using a cross-over design. This would allow exploiting variation in the data between classes and reduce the influence of timing. Finally, our study design only allowed us to analyze variation within classes over time. We, therefore, did not analyze variation between classes, although we observed some differences such as lower CO_2_ levels and transmission in School 2. These differences may be explained by school-specific factors not measured in our study.

In conclusion, using epidemiological, environmental, and molecular data, our study suggests that considerable transmission of SARS-CoV-2 occurred in the participating schools. General face mask wearing was associated with reduced SARS-CoV-2 transmission and prevented infections. The effectiveness of interventions was supported by significant decreases in the concentration of aerosols. Taken together, our results suggest that infection control measures can reduce the transmission of respiratory infections in school rooms. Future studies may use our multiple-measurement approach to assess the effectiveness of infection control measures in reducing the transmission of respiratory infections. Ideally, these data should be collected routinely in sentinel schools, thus continuously monitoring transmission risks and alerting health authorities when infection control measures should be taken.

## Supporting information

S1 STROBE Checklist STROBE Checklist. (PDF)

S1 Appendix Supplementary information. Text A. Details on laboratory and molecular analyses. Text B. Summary of case and molecular data. Text C. Longitudinal case data. Text D. Probabilistic simulation of case data. Text E. Estimating transmission and the effects of infection control measures. Text F. Model parameter estimation. Text G. Detailed results from transmission model. Text H. Estimating changes in particle concentrations. Text I. Computing rebreathed air volume and ventilation rate. Text J. Results for changes in environmental variables. Text K. Modeling transmission risk of SARS-CoV-2 using a modified Wells–Riley equation. Fig A. Proportion of suspected cases being actual cases of COVID-19. Fig B. Empirical and fitted distribution for the delay from symptom onset to absence. Fig C. Comparison of reported and estimates cases of COVID-19. Fig D. Prior for the probability of getting infected without interventions. Fig E. Choices of prior for incubation period. Fig F Estimated incidence over time. Fig G. Model- and simulation-based estimates of the number of COVID-19 cases. Fig H. Estimated number of avoided infections with interventions. Fig I. Boxplot of environmental variables by school and study condition. Fig J. Estimated transmission risk using a modified Wells–Riley equation. Table A. Reported cases of COVID-19, saliva, and airborne samples in School 1. Table B. Reported cases of COVID-19, saliva, and airborne samples in School 2. Table C. Overview of the study population, number of COVID-19 cases, and person-days of absences in each study class. Table D. List of confirmed and suspected cases over the study period in School 1. Table E. List of confirmed and suspected cases over the study period in School 2. Table F. Prior choices for model parameters. Table G. Estimation results from transmission model. Table H. Estimated reduction in aerosol and particle concentrations with interventions.
(PDF)

## Abbreviations

CrI: credible interval
COVID-19,: Coronavirus Disease 2019
CT: cycle threshold
MCMC: Markov chain Monte Carlo
NUTS: No-U-Turn Sampler
SARS-CoV-2: Severe Acute Respiratory Syndrome Coronavirus 2
VGL: viral genome load
VTM: viral transport medium
