# Supplementary material for: SARS-CoV-2 transmission with and without mask wearing or air cleaners in schools in Switzerland: A modeling study of epidemiological, environmental, and molecular data
Source: PLoS Med. 2023 May 18;20(5):e1004226. doi: 10.1371/journal.pmed.1004226 (PMC10194935; doi:10.1371/journal.pmed.1004226)
Supplement: S1 STROBE Checklist — (PDF) [file pmed.1004226.s001.pdf]

S1 Checklist. STROBE Checklist

STROBE Statement—checklist of items that should be included in reports of observational studies

	Item No	Recommendation	Section (paragraph no)
**Title and abstract**	1	(*a*) Indicate the study's design with a commonly used term in the title or the abstract	Title
		(*b*) Provide in the abstract an informative and balanced summary of what was done and what was found	Abstract – Methods and Findings
**Introduction**			
Background/rationale	2	Explain the scientific background and rationale for the investigation being reported	Introduction (paragraphs 1-2)
Objectives	3	State specific objectives, including any prespecified hypotheses	Introduction (paragraph 3)
**Methods**			
Study design	4	Present key elements of study design early in the paper	Methods – Study setting and design (paragraph 1); Methods – Study interventions (paragraph 1)
Setting	5	Describe the setting, locations, and relevant dates, including periods of recruitment, exposure, follow-up, and data collection	Methods – Study setting and design (paragraph 1)
Participants	6	(*a*) *Cohort study*—Give the eligibility criteria, and the sources and methods of selection of participants. Describe methods of follow-up<br>*Case-control study*—Give the eligibility criteria, and the sources and methods of case ascertainment and control selection. Give the rationale for the choice of cases and controls<br>*Cross-sectional study*—Give the eligibility criteria, and the sources and methods of selection of participants	Methods – Study setting and design (paragraph 1); Methods – Ethics statement (paragraph 1)
		(*b*) *Cohort study*—For matched studies, give matching criteria and number of exposed and unexposed<br>*Case-control study*—For matched studies, give matching criteria and the number of controls per case	
Variables	7	Clearly define all outcomes, exposures, predictors, potential confounders, and effect modifiers. Give diagnostic criteria, if applicable	Methods – Data collection (paragraphs 1-3)
Data sources/ measurement	8*	For each variable of interest, give sources of data and details of methods of assessment (measurement). Describe comparability of	Methods – Data collection (paragraphs 1-3); Methods – Laboratory and molecular

		assessment methods if there is more than one group	analyses (paragraphs 1); Texts A and I in S1 Appendix
Bias	9	Describe any efforts to address potential sources of bias	Methods – Statistical analyses and modeling (paragraph 1); Text D in S1 Appendix; Results – Analysis of aerosol and particle concentrations (paragraph 1)
Study size	10	Explain how the study size was arrived at	Methods – Study design and setting (paragraph 1); Results (paragraph 1); Table 1; Tables S1 and S2 in S1 Appendix
Quantitative variables	11	Explain how quantitative variables were handled in the analyses. If applicable, describe which groupings were chosen and why	Methods – Laboratory and molecular analyses (paragraph 1); Methods – Statistical analyses and modeling (paragraphs 1-2); Text C in S1 Appendix
Statistical methods	12	(*a*) Describe all statistical methods, including those used to control for confounding	Methods – Statistical analyses and modeling (paragraphs 1-5)
		(*b*) Describe any methods used to examine subgroups and interactions	
		(*c*) Explain how missing data were addressed	Methods – Statistical analyses and modeling (paragraph 1)
		(*d*) *Cohort study*—If applicable, explain how loss to follow-up was addressed<br>*Case-control study*—If applicable, explain how matching of cases and controls was addressed<br>*Cross-sectional study*—If applicable, describe analytical methods taking account of sampling strategy	
		(*e*) Describe any sensitivity analyses	Results – Estimating transmission risk of SARS-CoV-2 (paragraph 1)

Continued on next page

**Results**			
Participants	13*	(a) Report numbers of individuals at each stage of study—eg numbers potentially eligible, examined for eligibility, confirmed eligible, included in the study, completing follow-up, and analysed	Results (paragraph 1); Table 1; Tables 1-4 in S1 Appendix
		(b) Give reasons for non-participation at each stage	
		(c) Consider use of a flow diagram	
Descriptive data	14*	(a) Give characteristics of study participants (eg demographic, clinical, social) and information on exposures and potential confounders	Results (paragraph 1); Tables 1-4 in S1 Appendix
		(b) Indicate number of participants with missing data for each variable of interest	Tables 3-4 in S1 Appendix
		(c) *Cohort study*—Summarise follow-up time (eg, average and total amount)	
Outcome data	15*	*Cohort study*—Report numbers of outcome events or summary measures over time	Results (paragraph 1); Table 1; Tables 1-4 in S1 Appendix
		Case-control study—Report numbers in each exposure category, or summary measures of exposure	Tables 1-2 in S1 Appendix
		Cross-sectional study—Report numbers of outcome events or summary measures	
Main results	16	(*a*) Give unadjusted estimates and, if applicable, confounder-adjusted estimates and their precision (eg, 95% confidence interval). Make clear which confounders were adjusted for and why they were included	Results (paragraph 1); Results – Molecular analyses (paragraph 1); Results – Analysis of aerosol and particle concentrations (paragraph 1); Results – Estimating transmission risk of SARS-CoV-2 (paragraph 1); Methods – Statistical analyses and modeling (paragraphs 2, 3); Texts E and H in S1 Appendix
		(*b*) Report category boundaries when continuous variables were categorized	Methods – Data collection (paragraph 1); Text C in S1 Appendix
		(*c*) If relevant, consider translating estimates of relative risk into absolute risk for a meaningful time period	Methods – Statistical analyses and modeling (paragraph 4); Results – Estimating transmission risk of SARS-CoV-2 (paragraph 1); Figure 1b
Other analyses	17	Report other analyses done—eg analyses of subgroups and interactions, and sensitivity analyses	Results – Estimating transmission risk of SARS-CoV-2 (paragraph 1); Text K in S1 Appendix
**Discussion**			
Key results	18	Summarise key results with reference to study objectives	Discussion (paragraph 1)
Limitations	19	Discuss limitations of the study, taking into account sources of potential bias or imprecision. Discuss both direction and magnitude of any potential bias	Discussion (paragraph 1)

Interpretation	20	Give a cautious overall interpretation of results considering objectives, limitations, multiplicity of analyses, results from similar studies, and other relevant evidence	Discussion (paragraphs 2-5)
Generalisability	21	Discuss the generalisability (external validity) of the study results	Discussion (paragraph 7)
**Other information**			
Funding	22	Give the source of funding and the role of the funders for the present study and, if applicable, for the original study on which the present article is based	Meta data

*Give information separately for cases and controls in case-control studies and, if applicable, for exposed and unexposed groups in cohort and cross-sectional studies.

Note: An Explanation and Elaboration article discusses each checklist item and gives methodological background and published examples of transparent reporting. The STROBE checklist is best used in conjunction with this article (freely available on the Web sites of PLoS Medicine at http://www.plosmedicine.org/, Annals of Internal Medicine at http://www.annals.org/, and Epidemiology at http://www.epidem.com/). Information on the STROBE Initiative is available at www.strobe-statement.org.
