# Supplementary material for: SARS-CoV-2 transmission with and without mask wearing or air cleaners in schools in Switzerland: A modeling study of epidemiological, environmental, and molecular data
Source: PLoS Med. 2023 May 18;20(5):e1004226. doi: 10.1371/journal.pmed.1004226 (PMC10194935; doi:10.1371/journal.pmed.1004226)
Supplement: S1 Appendix — Text A. Details on laboratory and molecular analyses. Text B. Summary of case and molecular data. Text C. Longitudinal case data. Text D. Probabilistic simulation of case data. Text E. Estimating transmission and the effects of infection control measures. Text F. Model parameter estimation. Text G. Detailed results from transmission model. Text H. Estimating changes in particle concentrations. Text I. Computing rebreathed air volume and ventilation rate. Text J. Results for changes in environmental variables. Text K. Modeling transmission risk of SARS-CoV-2 using a modified Wells–Riley equation. Fig A. Proportion of suspected cases being actual cases of COVID-19. Fig B. Empirical and fitted distribution for the delay from symptom onset to absence. Fig C. Comparison of reported and estimates cases of COVID-19. Fig D. Prior for the probability of getting infected without interventions. Fig E. Choices of prior for incubation period. Fig F Estimated incidence over time. Fig G. Model- and simulation-based estimates of the number of COVID-19 cases. Fig H. Estimated number of avoided infections with interventions. Fig I. Boxplot of environmental variables by school and study condition. Fig J. Estimated transmission risk using a modified Wells–Riley equation. Table A. Reported cases of COVID-19, saliva, and airborne samples in School 1. Table B. Reported cases of COVID-19, saliva, and airborne samples in School 2. Table C. Overview of the study population, number of COVID-19 cases, and person-days of absences in each study class. Table D. List of confirmed and suspected cases over the study period in School 1. Table E. List of confirmed and suspected cases over the study period in School 2. Table F. Prior choices for model parameters. Table G. Estimation results from transmission model. Table H. Estimated reduction in aerosol and particle concentrations with interventions. (PDF) [file pmed.1004226.s002.pdf]

S1 Appendix

SARS-CoV-2 transmission with and without mask wearing or air cleaners in schools in Switzerland: A modeling study of epidemiological, environmental, and molecular data

Nicolas Banholzer[1‡], Kathrin Zürcher[1‡], Philipp Jent[2], Pascal Bittel[3], Lavinia Furrer[3], Matthias Egger[1], Tina Hascher[4], and Lukas Fenner[1*]

[1]Institute of Social and Preventive Medicine, University of Bern, Bern, Switzerland

[2]Department of Infectious Diseases, Inselspital, Bern University Hospital, University of Bern, Bern, Switzerland

[3]Institute of Infectious Diseases, University of Bern, Bern, Switzerland

[4]Institute of Educational Science, University of Bern, Bern, Switzerland

[*]Corresponding author: lukas.fenner@ispm.unibe.ch

[‡]These authors contributed equally to this work.

List of Texts

A Details on laboratory and molecular analyses 4

B Summary of case and molecular data 4

C Longitudinal case data 6

D Probabilistic simulation of case data 10

E Estimating transmission and the effects of infection control measures 14

E.1 Overall approach 14

E.2 Notation 14

E.3 Relating the number of new infections to the number of susceptibles and the presence of interventions 14

E.4 Relating the number susceptibles to the number of infections 15

E.5 Relating the number new observed cases to the number of new infections 16

E.6 Taking into account transmission outside school days 16

E.7 Seeding phase 17

E.8 Adjusting for absences and community transmission 17

E.9 Choice of priors for the probability of getting infected without interventions 17

E.10 Choice of priors for the effects of interventions 19

E.11 Choice of priors for the incubation period 19

E.12 Choice of prior distributions (summary) 19

F Model parameter estimation 21

G Detailed results from transmission model 22

G.1 Estimation results 22

G.2 Estimated cumulative and new number of infections 24

G.3 Overall model fit 25

G.4 Estimated avoided infections for each generated dataset 26

H Estimating changes in particle concentrations **27**

I Computing rebreathed air volume and ventilation rate **28**

J Results for changes in environmental variables **29**

K Modeling transmission risk of SARS-CoV-2 using a modified Wells-Riley equation **31**

References **33**

List of Figures

A Proportion of suspected cases being actual cases of COVID-19 11
B Empirical and fitted distribution for the delay from symptom onset to absence . . . 12
C Comparison of reported and estimated cases of COVID-19 13
D Prior for the probability of getting infected without interventions 18
E Choices of prior for incubation period 19
F Estimated incidence over time 24
G Model- and simulation-based estimates of the number of COVID-19 cases 25
H Estimated number of avoided infections with interventions 26
I Boxplot of environmental variables by school and study condition 29
J Estimated transmission risk using a modified Wells-Riley equation 32

List of Tables

A Reported cases of COVID-19, saliva and airborne samples in School 1 4
B Reported cases of COVID-19, saliva and airborne samples in School 2 5
C Overview of the study population, number of COVID-19 cases, and person-days of absences in each study class. 5
D List of confirmed and suspected cases over the study period in School 1 6
E List of confirmed and suspected cases over the study period in School 2 8
F Prior choices for model parameters. 20
G Estimation results from transmission model 23
H Estimated reduction in aerosol and particle concentrations with interventions 30

Text A. Details on laboratory and molecular analyses

The Allplex RV Master Assay (Seegene, Seoul, South Korea) detects a panel of 19 respiratory viruses: SARS-CoV-2, Influenza A virus, Influenza B virus, Human respiratory syncytial virus A/B (RSV), Human metapneumovirus (MPV), Human adenovirus A/B/C/D/E/F (AdV), Human rhinovirus A/B/C (HRV), and Human parainfluenza virus 1/2/3/4 (PIV) in one single reaction. Viral genome load (VGL) of specimens was quantified (copies/L) by curve extrapolation derived from standardized ATCC quantitative genomic standard dilutions (ATCC-VR-3347D, LGC Standards, LGC Group UK). For the BioSpot-VIVAS air samples, the air concentration of the DNA was exactly calculated according to the airflow influx into the sampling device. The Limit of detection (LOD) of the assay SARS-CoV-2, IFA/IFB is $7.5\text{x}10^{0}$ TCID/ml (SARS-CoV-2), $3.7\text{x}10^{-1}$ TCID/ml (IFA), and 4.8x100 TCID/ml (100cp/ml) (IFB) respectively.

Text B. Summary of case and molecular data

Table A. By study condition (first row) and week (second row) in School 1, the average number of students in class per day (third row), number of confirmed and suspected cases of COVID-19 (rows 4-9), number of negative and positive saliva (rows 10-12) and airborne (rows 13-19) samples of SARS-CoV-2, and positive air samples on the filters of air cleaners (rows 20-21).

	**Mask mandate**		**No intervention**			**Air cleaner**		**Mean/Total**†
Study week	1	2	3	4	5	6	7	
No. of students	40	44	44	45	35	44	46	43
Confirmed	10	0	0	1	14	0	0	25
Positive test result	1	0	0	0	6	0	0	7
Isolation	9	0	0	1	8	0	0	18
Suspected	7	5	5	13	12	5	6	53
Known symptoms	4	2	1	2	6	3	0	18
Unknown symptoms	3	3	4	11	6	2	6	35
Saliva samples	21	44‡	26	29	20	18	15	173
Negative	20	42	26	27	14	17	15	161
Positive	1	2	0	2	6	1	0	12
Air samples	9	9	10	10	10	10	10	68
Coriolis	4	4	5	5	5	5	5	33
Vivas	5	5	5	5	5	5	5	35
Negative	9	9	10	8	10	10	8	64
Positive	0	0	0	2	0	0	2	4
Vivas: Copies/L	0	0	0	2.46	0	0	0.36	
Coriolis: Copies/L	0	0	0	0	0	0	0.12	
Positive air samples on filter 1								2
Positive air samples on filter 2								0

† "Mean" refers to the average number of students per day and "Total" to the total number of cases, saliva, and air samples over the study period, respectively.

‡ Saliva samples usually collected once per week, except in the second week where it was two times.

Table B. By study condition (first row) and week (second row) in School 2, the average number of students in class per day (third row), number of confirmed and suspected cases of COVID-19 (rows 4-9), number of negative and positive saliva (rows 10-12) and airborne (rows 13-19) samples of SARS-CoV-2, and positive air samples on the filters of air cleaners (rows 20-21).

	**Mask mandate**				**No intervention**	**Air cleaner**		**Mean/Total**†
Study week	1	2	3	4	5	6	7	
No. of students	33	31	33	31	32	31	29	31
Confirmed	2	0	0	1	0	5	2	10
Positive test result	2	0	0	1	0	5	2	10
Isolation	0	0	0	0	0	0	0	0
Suspected	1	5	2	3	4	3	2	20
Known symptoms	1	5	2	3	2	3	2	18
Unknown symptoms	0	0	0	0	2	0	0	2
Saliva samples	17	20‡	14	10	11	12	5	89
Negative	16	20	12	10	10	9	5	82
Positive	1	0	2	0	1	3	0	7
Air samples	9	9	8	10	8	9	9	62
Coriolis	4	4	4	5	4	4	5	30
Vivas	5	5	4	5	4	5	4	32
Negative	8	6	8	10	7	9	9	57
Positive	1	3	0	0	1	0	0	5
Vivas: Copies/L	1.27	2.68	0	0	2.07	0	0	
Coriolis: Copies/L	0	0.16	0	0	0	0	0	
Positive air samples on filter 1								4
Positive air samples on filter 2								3

† "Mean" refers to the average number of students per day and "Total" to the total number of cases, saliva, and air samples over the study period, respectively.

‡ Saliva samples usually collected once per week, except in the second week where it was two times.

Table C. Overview of the study population, number of COVID-19 cases, and person-days of absences in each study class.

	School 1			School 2		Total
Class	A	B	C	D	E	
**Students**	**24 (27%)**	**14 (16%)**	**14 (16%)**	**20 (22%)**	**18 (20%)**	**90 (100%)**
Sex						
↳ Female	11 (46%)	4 (29%)	5 (36%)	10 (50%)	9 (50%)	39 (43%)
↳ Male	13 (54%)	10 (71%)	9 (64%)	10 (50%)	9 (50%)	51 (57%)
Vaccination status						
↳ Vaccinated	10 (42%)	3 (21%)	3 (21%)	7 (35%)	4 (22%)	27 (30%)
↳ Not vaccinated	14 (58%)	11 (79%)	11 (79%)	13 (65%)	14 (68%)	63 (70%)
Recovery status						
↳ Recovered last year	6 (25%)	10 (71%)	9 (64%)	4 (20%)	5 (28%)	34 (38%)
↳ Not recovered	18 (75%)	4 (29%)	5 (36%)	16 (80%)	13 (72%)	56 (62%)
**Absent person-days**	**142 (22%)**	**111 (17%)**	**81 (13%)**	**221 (34%)**	**89 (14%)**	**644 (100%)**
↳ Isolation	52 (37%)	27 (24%)	30 (37%)	23 (10%)	15 (17%)	147 (23%)
↳ Sickness	17 (12%)	47 (42%)	31 (38%)	116 (53%)	36 (40%)	247 (38%)
↳ Quarantine	45 (31%)	4 (4%)	6 (8%)	5 (2%)	0 (0%)	60 (9%)
↳ Other	28 (20%)	33 (30%)	14 (17%)	77 (35%)	38 (43%)	190 (30%)
**COVID-19 cases**	**24 (22%)**	**33 (31%)**	**21 (19%)**	**16 (21%)**	**14 (13%)**	**108 (100%)**
↳ Confirmed	13 (54%)	6 (18%)	6 (29%)	7 (44%)	3 (21%)	35 (32%)
↳ Suspected	11 (46%)	27 (82%)	15 (71%)	9 (56%)	11 (79%)	73 (68%)

Text C. Longitudinal case data

For every school day, we collected information on the students being absent, i. e. their reason for being absent, whether they had symptoms related to COVID-19, and whether they had tested positive for COVID-19. The reason for being absent could not be obtained from some students who did not give informed consent. As a result, we distinguish between four types of cases of COVID-19:

- **Confirmed (positive test result)**: Absence due to a positive lab test result for COVID-19.
- **Confirmed (isolation)**: Absence due to isolation as a result from an infection with COVID-19, but without information on the lab test result.
- **Suspected (known symptoms)**: Absence due to sickness with at least one symptom related to COVID-19, i. e. fever, coughing, tiredness, loss of test or smell, sore throat, headache, aches and pains, diarrhoea, difficulty breathing or shortness of breath, stomach.
- **Suspected (unknown symptoms)**: Absence due to sickness with unknown symptoms, i. e. information on symptoms could not be obtained as no informed consent was given.

Tables D-E shows the list of confirmed and suspected cases of COVID-19 for School 1 and 2. The date of symptom onset was not always known or reported, but should be close or corresponding to the date of absence.

Table D. List of confirmed and suspected cases over the study period in School 1.

Class	Date of absence	Date of symptom onset	Type of case
A	01-24	01-21	Confirmed (positive test result)
B	01-24		Confirmed (isolation)
B	01-24		Confirmed (isolation)
A	01-24		Confirmed (isolation)
A	01-24		Confirmed (isolation)
C	01-24		Confirmed (isolation)
C	01-24		Confirmed (isolation)
B	01-24		Suspected (known symptoms)
B	01-25		Confirmed (isolation)
B	01-25		Suspected (known symptoms)
B	01-25		Suspected (unknown symptoms)

C	01-26		Confirmed (isolation)
B	01-26		Suspected (known symptoms)
B	01-27		Suspected (known symptoms)
C	01-28		Confirmed (isolation)
A	01-28		Suspected (unknown symptoms)
C	01-28		Suspected (unknown symptoms)
A	01-31		Suspected (unknown symptoms)
A	02-01		Suspected (unknown symptoms)
B	02-03		Suspected (known symptoms)
B	02-03		Suspected (known symptoms)
C	02-03		Suspected (unknown symptoms)
A	02-21		Suspected (unknown symptoms)
B	02-21		Suspected (known symptoms)
A	02-22		Suspected (unknown symptoms)
C	02-23		Suspected (unknown symptoms)
A	02-24		Suspected (unknown symptoms)
B	02-28		Suspected (known symptoms)
B	02-28		Suspected (unknown symptoms)
C	02-28		Suspected (unknown symptoms)
C	03-01		Suspected (unknown symptoms)
B	03-02		Suspected (unknown symptoms)
C	03-02		Suspected (unknown symptoms)
C	03-02		Suspected (unknown symptoms)
C	03-02		Suspected (unknown symptoms)
A	03-03		Suspected (unknown symptoms)
B	03-03		Suspected (unknown symptoms)
A	03-04		Confirmed (isolation)
A	03-04		Suspected (unknown symptoms)
A	03-04		Suspected (unknown symptoms)
B	03-04		Suspected (known symptoms)
B	03-07	03-07	Confirmed (positive test result)
A	03-07	03-04	Confirmed (positive test result)
A	03-07	03-06	Confirmed (positive test result)
A	03-07		Confirmed (isolation)
A	03-07		Confirmed (isolation)
B	03-07		Suspected (known symptoms)

C	03-07		Suspected (unknown symptoms)
C	03-08		Confirmed (isolation)
B	03-08		Suspected (known symptoms)
B	03-08		Suspected (known symptoms)
C	03-08		Suspected (unknown symptoms)
A	03-09	03-09	Confirmed (positive test result)
A	03-09		Confirmed (positive test result)
A	03-09		Confirmed (isolation)
B	03-09		Suspected (known symptoms)
B	03-10		Confirmed (isolation)
A	03-10		Confirmed (isolation)
C	03-10		Confirmed (isolation)
B	03-10		Suspected (known symptoms)
B	03-10		Suspected (known symptoms)
A	03-11	03-11	Confirmed (positive test result)
B	03-11		Confirmed (isolation)
A	03-11		Suspected (unknown symptoms)
C	03-11		Suspected (unknown symptoms)
C	03-11		Suspected (unknown symptoms)
C	03-11		Suspected (unknown symptoms)
B	03-14		Suspected (known symptoms)
B	03-14		Suspected (known symptoms)
B	03-15		Suspected (known symptoms)
B	03-17		Suspected (unknown symptoms)
B	03-18		Suspected (unknown symptoms)
B	03-21		Suspected (unknown symptoms)
B	03-21		Suspected (unknown symptoms)
A	03-23		Suspected (unknown symptoms)
C	03-23		Suspected (unknown symptoms)
C	03-23		Suspected (unknown symptoms)
B	03-25		Suspected (unknown symptoms)

Table E. List of confirmed and suspected cases over the study period in School 2.

Class	Date of absence	Date of symptom onset	Type of case
E	01-24	01-24	Confirmed (positive test result)

D	01-26	01-26	Suspected (known symptoms)
D	01-27	01-26	Confirmed (positive test result)
D	01-31	01-31	Suspected (known symptoms)
E	02-02	02-02	Suspected (known symptoms)
D	02-03	02-03	Suspected (known symptoms)
E	02-04	02-04	Suspected (known symptoms)
E	02-04	02-04	Suspected (known symptoms)
D	02-14		Suspected (known symptoms)
E	02-14		Suspected (known symptoms)
D	02-21		Confirmed (positive test result)
D	02-21	02-21	Suspected (known symptoms)
D	02-21	02-21	Suspected (known symptoms)
E	02-22	02-22	Suspected (known symptoms)
D	03-02	03-02	Suspected (known symptoms)
D	03-03	03-03	Suspected (known symptoms)
E	03-03		Suspected (unknown symptoms)
E	03-03	03-03	Suspected (unknown symptoms)
E	03-07		Suspected (known symptoms)
E	03-08	03-08	Confirmed (positive test result)
D	03-08		Confirmed (positive test result)
E	03-08		Suspected (known symptoms)
D	03-09		Confirmed (positive test result)
E	03-09	03-09	Suspected (known symptoms)
D	03-10	03-10	Confirmed (positive test result)
D	03-11	03-11	Confirmed (positive test result)
E	03-14	03-13	Confirmed (positive test result)
D	03-14	03-14	Confirmed (positive test result)
D	03-14		Suspected (known symptoms)
E	03-16		Suspected (known symptoms)

Text D. Probabilistic simulation of case data

Our epidemiological data is incomplete and subject to uncertainty. The reason is that a) we have a considerable number of suspected but unconfirmed cases and b) we do not know the date of symptom onset for many cases. Regarding a), it is likely that a considerable proportion of suspected cases are actual cases of COVID-19, but we do not know which of the suspected cases are actual cases. Regarding b), if the date of symptom onset is unknown, we could assume that it corresponds to the date of absence, but analysis of complete cases suggests that the date of symptom onset may sometimes precede the date of absence (i. e. students have been in school despite having symptoms). We would want to take uncertainty about a) and b) into account when estimating transmission from the observed number of new cases. As a consequence, we use a probabilistic simulation approach where we generate $D = 100$ datasets for the number of new cases by date of symptom onset.

In the first step, we simulate the number of suspected cases being actual cases. For this, we assume that each student may only be infected once over the seven-week study period. Since the total number of confirmed and suspected cases exceed the number of students in some classes, it is clear that not all suspected cases can be actual cases of COVID-19. Let C_{jT}^{CF}, C_{jT}^{SU}, and $C_{jT}^{\text{CF+SU}}$ be the total number of confirmed, suspected and confirmed plus suspected cases in class $j = 1, \ldots, J$ at the end of the study period T, respectively. Further, let p_j be the proportion of suspected cases being actual cases of COVID-19 in class j. This proportion ranges from l_j to u_j, where

$$l_j = 0, \tag{1}$$

$$u_j = \frac{\max(Y_j - C_{jT}^{\text{CF}}, C_{jT}^{\text{SU}})}{C_{jT}^{\text{CF+SU}}} \ . \tag{2}$$

We assume that p_j follows a truncated normal distribution, i. e.

$$p_j \sim \text{Normal}_{l_j}^{u_j} \left(\mu = \frac{u_j - l_j}{2}, \sigma = \frac{u_j - l_j}{2 \cdot \Phi^{-1}(0.95)} \right) \tag{3}$$

where $\Phi^{-1}(\alpha)$ is the inverse of the cumulative standard normal distribution function at the α% quantile. Fig. A shows the distributions for $p_j, \ldots, p_J$ based on our data. In each simulation, we draw a value for $p_j, \ldots, p_J$ and then sample suspected cases with equal probability p_j in each class.

The sample of suspected cases is combined with the number of confirmed cases into one series of the observed number of new cases of COVID-19.

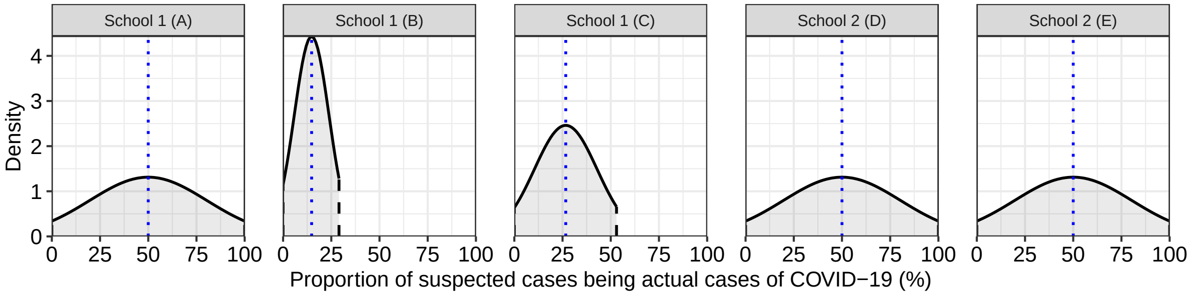

Fig A. Distribution for the proportion of suspected cases being actual cases of COVID-19 in each class.

In the second step, we simulate the date of symptom onset. For this, we use the empirical distribution for the delay between date of symptom onset and date of absence of the cases where the date of symptom onset has been reported (Fig. B). We fit an exponential curve through these data points and compute the probability for the delays as the fitted frequency for each day divided by the total frequency. In each simulation, we use this probability distribution to sample the delay between the unknown dates of symptom onset and the known dates of absence and, based on that, compute the date of symptom onset.

Both simulation steps are performed 100 times to generate $D = 100$ datasets. Fig. C shows the reported number of confirmed and suspected cases in comparison with the estimated number of cases after probabilistic simulation over time in each class.

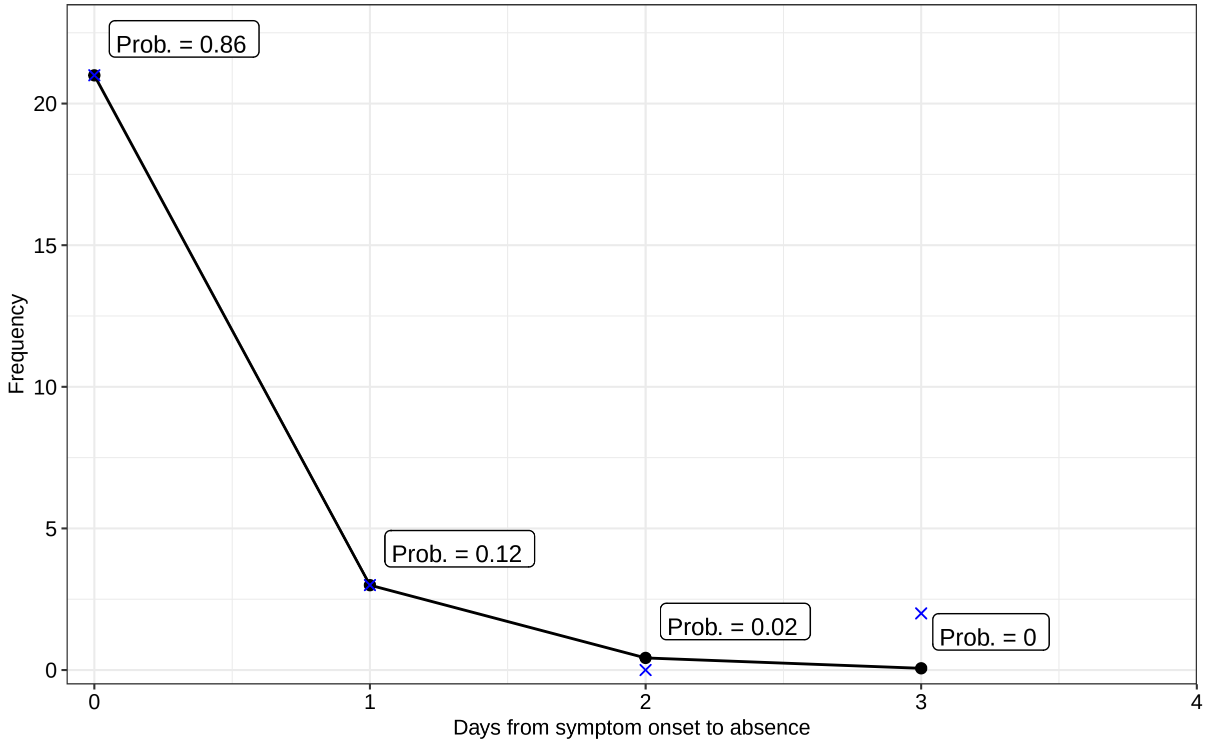

Fig B. Empirical (blue) and fitted (black) distribution for the delay from symptom onset to absence. Labels refer to the probability for each day of delay, which is computed by dividing the frequency from the fitted exponential curve by the total frequency.

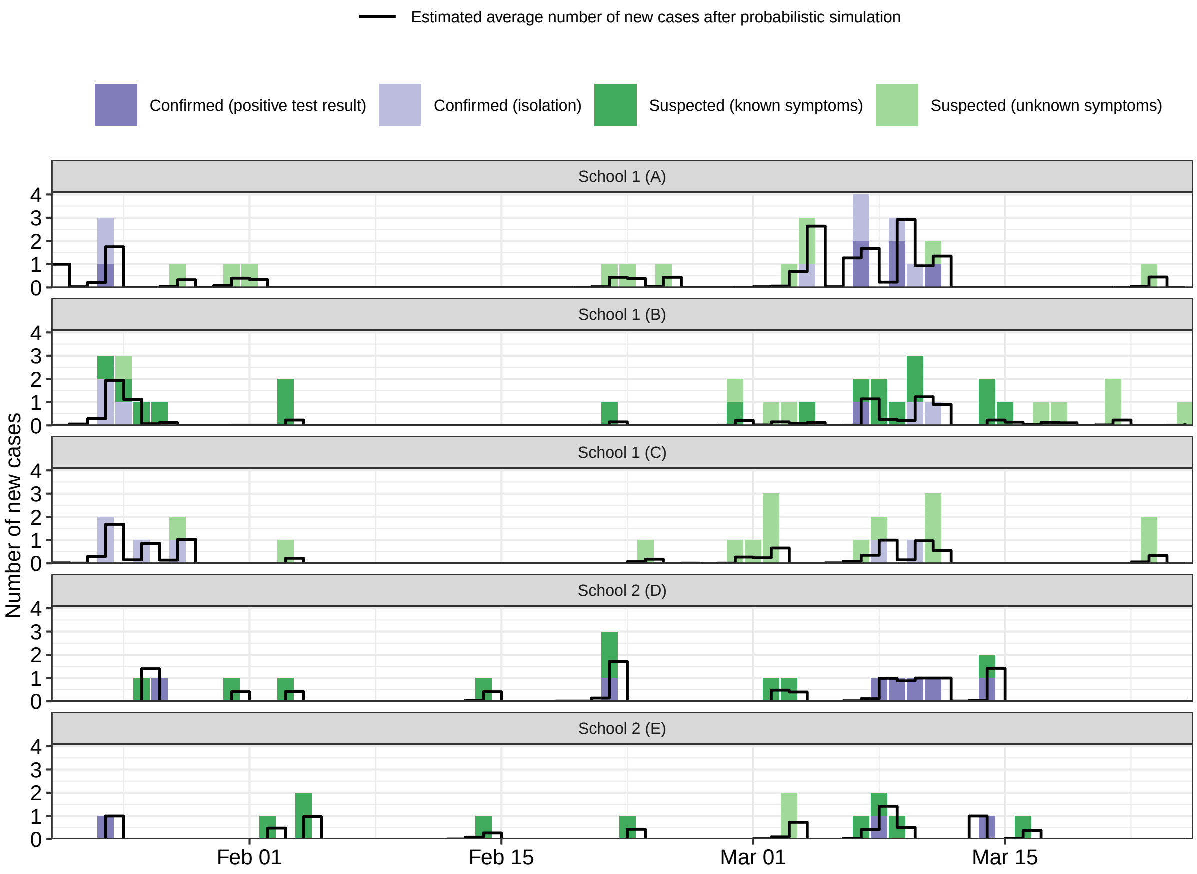

Fig C. Reported number of new confirmed (purple bars) and suspected (green bars) cases in comparison with the estimated number of new cases after probabilistic simulation (black line) over time in each class.

Text E. Estimating transmission and the effects of infection c

E.1 Overall approach

The aim is to estimate transmission of SARS-CoV-2 and the effects of infection control measures (mask mandates and air cleaners) on the number of new infections with COVID-19. To estimate transmission, our model links two unobserved quantities (the number of new infections and susceptible students) with one observed quantity (the number of new cases among students). Specifically, we link the number of new cases to the number of new infections in the previous days. We then formulate the daily proportion of susceptible students getting infected as a function of the interventions in place. The effects of interventions are estimated with a Bayesian approach, which requires the specification of prior distributions for all model parameters.

E.2 Notation

j	class
t	days since start of study period
Y_j	total number of students in class j (unobserved)
N_{jt}	number of new cases among students in class j at day t (observed)
I_{jt}	number of new infections in class j at day t (unobserved)
S_{jt}	number of susceptibles in class j at day t (unobserved)

E.3 Relating the number of new infections to the number of susceptibles and the presence of interventions

A proportion of the susceptible students P_{jt} gets infected in class $j = 1, \ldots, J$ at each day $t = 1, \ldots, T$

$$I_{jt} = S_{jt} P_{jt} \ . \tag{4}$$

This proportion (or probability) can be estimated using a logit link function

$$\Phi_{jt} = \log\left(\frac{P_{jt}}{1-P_{jt}}\right) \tag{5}$$

$$\text{logit}^{-1}(\Phi_{jt}) = \frac{\exp(\Phi_{jt})}{1+\exp(\Phi_{jt})} = P_{jt} \ . \tag{6}$$

In the absence of interventions, the probability of getting infected is constant, i. e. $\Phi_{jt} = \beta_j$. It can only change at times when interventions are in place. Let θ_j^M be the effect of mask mandates and let θ_j^A be the effect of air cleaners in class j, then

$$\Phi_{jt} = \beta_j + \theta_j^M M_{jt} + \theta_j^A A_{jt}, \tag{7}$$

where M_{jt} and A_{jt} are binary variables indicating whether mask mandates and air cleaners are in place in class j at day t.

E.4 Relating the number susceptibles to the number of infections

The daily number of susceptibles S_{jt} are computed as the difference between the total number of students Y_j and the cumulative number of infections C_{jt} in the previous days

$$S_{jt} = Y_j - C_{jt} = Y_j - \sum_{s<t} I_{js} \ . \tag{8}$$

We assume that all students are susceptible at the beginning of the study period, despite the fact that some students were vaccinated or already infected with COVID-19 prior to the beginning of our study. Note that varying susceptibility before study onset can be subsumed in the class-specific transmission rate without interventions β_j.

E.5 Relating the number new observed cases to the number of new infections

The expected number μ of new cases N_{jt} in country j at day t can be derived from the number of new infections in the previous days as

$$\mu^{N_{jt}} = \sum_{s<t} I_{js} \cdot p_{\text{IN}}(t-s) \ , \tag{9}$$

where $p_{\text{IN}}(t)$ denotes the probability distribution of the incubation period, i. e. the probability that a new infected subject reports symptoms at day t after the infection. This distribution is estimated from our data as part of fitting the overall model using an informative prior (Section E.11).

The observed number of new cases are modeled to follow a negative binomial distribution, i. e.

$$N_{jt} \sim \text{NegBinom}\left(\mu^{N_{jt}}, \sigma^{N_{jt}}\right) \tag{10}$$

with mean $\mu^{N_{jt}}$, standard deviation $\sigma^{N_{jt}} = \sqrt{\mu^{N_{jt}}\left(1+\frac{\mu^{N_{jt}}}{\phi^N}\right)}$, and an overdispersion parameter ϕ^N.

E.6 Taking into account transmission outside school days

Absences were reported on school days but symptom onset could have occurred on the weekend, e. g. a student absent on Monday due to COVID-19 may report Saturday as the day of symptom onset. Some cases may still have gone unreported because the date of absence was the same as the date of symptom onset for the majority of cases (see Fig. B). Therefore, we distinguish between the transmission rate during and outside schools days, i. e.

$$\beta_j = \alpha_j + \omega, \tag{11}$$

where α_j and $\alpha_j + \omega$ are the logit of the probability of getting infected without interventions during and outside school days, respectively. We estimate α_j and ω from data using informative priors (Section E.9). We further assume that transmission outside school days is the same regardless of whether they are weekend or vacation days, but we do not incorporate vacation days into the model likelihood. That is, during vacation, the modeling of N_{jt} is ignored but I_{jt} and S_{jt} are still computed.

E.7 Seeding phase

A few cases were reported in the first days of the study period, indicating that the corresponding infections could have occurred before the start of the study. Therefore, we seed the number of new infections and cases before the study period by initiating our model 12 days (double the average incubation period) prior to the start of the study. Before this day, we assume there have been no infections and all students are susceptible.

E.8 Adjusting for absences and community transmission

The probability of getting infected may be influenced by factors other than interventions. We account for two such factors. First, we adjust our estimates for the daily proportion of students absent from class. We expect this proportion to decrease the probability of getting infected because absent students cannot infect classmates and, if staying home, they are also less likely to get infected themselves (if not already infected). Second, we adjust our estimates for the risk of community transmission, which we proxy with the average of the median effective reproduction number for the canton of Solothurn, Switzerland[1]. We expect higher community transmission to increase the probability of getting infected. The modeling parameters of these factors are denoted by $\boldsymbol{\gamma}$.

E.9 Choice of priors for the probability of getting infected without interventions

We choose informative priors for the logit of the daily probability of getting infected in the absence of interventions $\beta_j = \alpha_j + \omega$. First, our prior for α_j is derived from the share of susceptibles by the end of the study period S_{jT}/Y_j, i. e.

$$S_{jT}/Y_j = (1 - \text{logit}^{-1}(\alpha_j))^{T_{\text{school}}}, \tag{12}$$

where T_{school} is the number of school days (excluding weekends and vacation), and solving for α_j

$$\text{logit}^{-1}(\alpha_j) = 1 - \exp\left(\frac{\log(S_{jT}/Y_j)}{T_{\text{school}}}\right) . \tag{13}$$

Note that S_{jT}/Y_j and correspondingly $\text{logit}^{-1}(\alpha_j)$ will be different for each of our probabilistically generated datasets. Fig. Da shows the daily proportion of susceptibles getting infected based on the distributions for the proportion of suspected cases being actual cases of COVID-19 (see Fig. A). The corresponding distribution for α_j is shown in Fig. Db. We choose a prior for $\alpha_j \sim \text{Student-t}(\nu = 3, \mu = -4.1, \sigma = 1.6)$ with wider tails than the empirical distribution to accommodate the fact that α_j may be smaller or larger depending on the effects of interventions and adjustment variables.

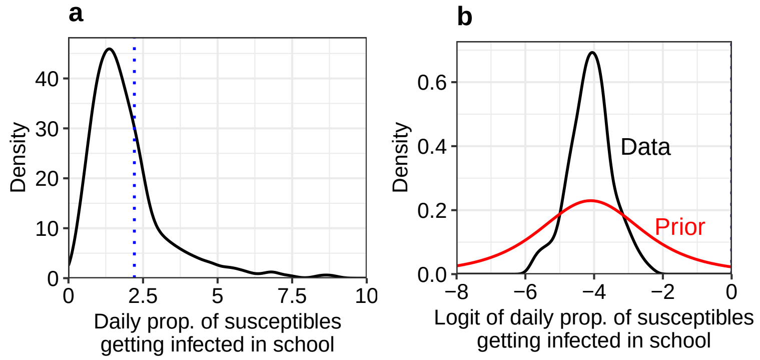

Fig D. Prior for the probability of getting infected without interventions. **(a)** Empirical distribution for the daily proportion of susceptibles getting infected across classes. The dotted blue line indicates the mean. **(b)** Empirical distribution for the logit of the daily proportion of susceptibles getting infected. The dotted blue line indicates the empirical mean. The corresponding choice of prior for the probability of getting infected without interventions α is hown in red.

Second, we choose a prior for $\omega \sim \text{Normal}(\mu = \log 0.7, \sigma = 0.2)$ that encodes our prior belief that students will have fewer indoor contacts outside school days and thus lower odds of getting infected. In addition, this informative prior also considers potential underreporting of cases on weekends (see Section E.6).

E.10 Choice of priors for the effects of interventions

We consider that the mandates may not have been equally effective in all classes. Therefore, we model the effects of mask mandates as $\theta_j^M = \text{Normal}(\theta^M, \tau)$, where $\theta^M \sim \text{Student-t}(\nu = 7, \mu = 0, \sigma = 2.5)$ is the average effect across classes and $\tau \sim \text{Student-t}^+(\nu = 5, \mu = 0, \sigma = 1)$ is the variation in the effect between classes.

Air cleaners only applied to three classes and were installed towards the end of the study period when most students may have already been infected. Therefore, we do not estimate variation in the effect between classes but only the average effect. In addition, we choose a prior leaning towards smaller effects, i. e. $\theta^A \sim \text{Student-t}(\nu = 7, \mu = 0, \sigma = 1)$.

E.11 Choice of priors for the incubation period

We estimate the distribution of the incubation period from our data as part of fitting the overall model. Our prior choices for the parameters of this distribution are informed by a meta-analysis[2], which reported a Lognormal distribution with $\log \mu \sim \text{Normal}(1.63, 0.06)$ and $\log \sigma \sim \text{Normal}^+(0.50, 0.05)$. The prior for p_{IN} is shown in Fig. E. Note that p_{IN} is discretized via $p_{\text{IN}}(s) = \int_0^{0.5} p_{\text{IN}}(\tau)\, \mathrm{d}\tau$ for $s = 0$ and $p_{\text{IN}}(s) = \int_{s-0.5}^{s+0.5} p_{\text{IN}}(\tau)\, \mathrm{d}\tau$ for $s > 0$, where $p_{\text{IN}}(\tau) \sim \text{Lognormal}(\mu, \sigma)$ is the density of the lognormal distribution.

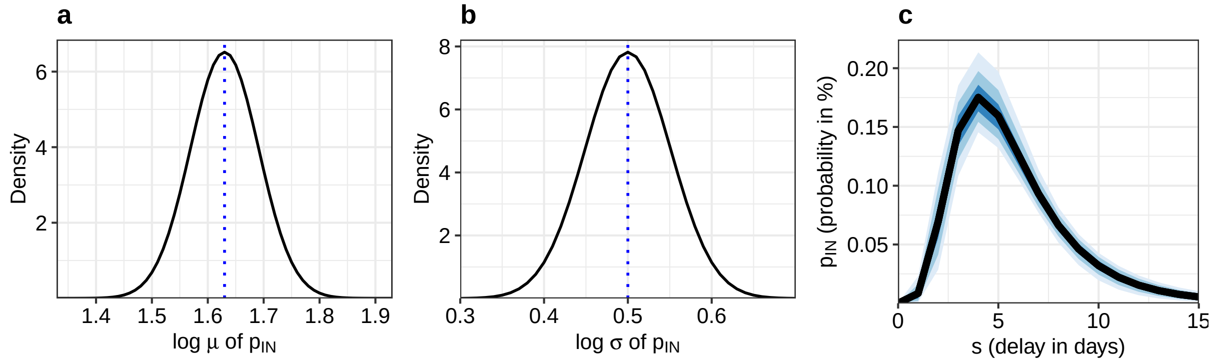

Fig E. Choices of prior for incubation period. **(a)** Prior for parameter $\log \mu$. **(a)** Prior for parameter $\log \sigma$. **(c)** Resulting prior p_{IN} (posterior mean as line and 50%, 80%, and 95%-quantile as shaded area).

E.12 Choice of prior distributions (summary)

Table F provides an overview of the model parameters together with the prior choices. If not tailored to the specifics of the model, our choices are informed by recommendations on the choice of priors

Parameter	Notation	(Hyper-)Prior
Effects of mask mandates	θ_j^M	$\text{Normal}(\theta^M, \tau)$
	θ^M	$\text{Student-t}(\nu = 7, \mu = 0, \sigma = 2.5)$
	τ	$\text{Student-t}^+(\nu = 5, \mu = 0, \sigma = 1)$
Effects of air cleaners	θ^A	$\text{Student-t}(\nu = 7, \mu = 0, \sigma = 1)$
Prob. of getting infected w/o interventions (logit)	β_j	$\beta_j = \alpha_j + \omega$
Prob. of getting infected during school (logit)	α_j	$\text{Student-t}(\nu = 3, \mu = -4.1, \sigma = 1.6)$
Weekend effect	ω	$\text{Normal}(\mu = \log 0.7, \sigma = 0.2)$
Overdispersion	ϕ^N	$\phi^N = \left(\frac{1}{\xi^N}\right)^2$
	ξ^N	$\text{Normal}^+(\mu = 0, \sigma = 1)$
Time from infection to new case	p_{IN}	$\text{Lognormal}(\log \mu, \log \sigma)$
	μ	$\text{Normal}(\mu = 1.63, \sigma = 0.06)$
	σ	$\text{Normal}^+(\mu = 0.50, \sigma = 0.05)$
Adjustments	γ	$\text{Student-t}(\nu = 7, \mu = 0, \sigma = 2.5)$

Table F. Prior choices for model parameters.

from the Stan Development Team[3].

Text F. Model parameter estimation

All model parameters are estimated with a Bayesian approach. Specifically, Markov chain Monte Carlo (MCMC) sampling is used as implemented by the Hamiltonian Monte Carlo algorithm with the No-U-Turn Sampler (NUTS) from Stan 2.21.0[4]. If not stated otherwise, we report posterior means and credible intervals (CrIs) based on the 50%, 80%, and 95% quantiles of the posterior samples, respectively.

Each model is estimated with 4 Markov chains and 2,000 iterations of which the first 1,000 iterations are discarded as part of the warm-up. Estimation power is evaluated via the effective sample size ESS and convergence of the Markov chains is evaluated with the Gelman-Rubin convergence diagnostic ($\hat{R}$).

Text G. Detailed results from transmission model

G.1 Estimation results

Table G presents the posterior mean, credible intervals and model diagnostics for all model parameters as the average across our 100 generated datasets (Section D). See the main paper for a discussion of the effects of interventions. Here we briefly discuss some of the additional model parameters.

The effective sample size (ESS) and the Gelman-Rubin convergence diagnostic ($\hat{R}$) indicate good estimation power. It further suggests that the Markov chains converged.

The estimate for τ indicates that there is variation in the effects of mask mandates between classes. However, the credible intervals of θ_j^M all include zero, indicating that there is only mild deviation of the class-specific estimates from their cross-class average estimate θ^M.

The mean estimate for the proportion of students being absent is negative, indicating that higher proportion of absences decrease the probability of getting infected. In contrast to that, the estimate for the reproduction number in the community is positive, indicating that higher community transmission increases the probability of getting infected. Both estimates are in line with our hypothesized effect, although it should be noted that both estimates have large credible intervals including zero.

The overdispersion parameter (ϕ) cannot be precisely estimated, but the credible intervals indicate rather small overdispersion ($\phi \to \infty$).

The estimates for the logit of the probability of getting infected during school days without interventions (α) are larger than the prior mean. This is due to the reduction in transmission from mask mandates. The weekend effect (ω) is negative, but smaller than the mean of our informative prior, indicating that the likelihood suggests more comparable transmission on weekends.

The posterior distribution of the parameters of the incubation period ($\mu^{p_{IN}}$ and $\sigma^{p_{IN}}$) are close to their prior, indicating that the data is not informative about the incubation period.

Table G. Estimation results from transmission models across the 100 generated datasets for the number of new cases of COVID-19.

Parameter	Mean	Lower 95%-CrI	Upper 95%-CrI	$\hat{R}$	ESS
ϕ	6401.17	0.58	138.33	1.01	1622.95
$\alpha_{\text{School 1 (A)}}$	−2.76	−4.08	−1.35	1.02	1342.87
$\alpha_{\text{School 1 (B)}}$	−2.71	−4.12	−1.25	1.02	1529.45
$\alpha_{\text{School 1 (C)}}$	−2.53	−4.00	−1.06	1.01	1411.09
$\alpha_{\text{School 2 (D)}}$	−2.82	−4.17	−1.36	1.02	1556.06
$\alpha_{\text{School 2 (E)}}$	−3.21	−4.61	−1.66	1.01	1691.10
ω	−0.31	−0.64	0.01	1.01	2738.59
$\mu^{p_{IN}}$	1.65	1.55	1.75	1.02	2942.18
$\sigma^{p_{IN}}$	0.50	0.42	0.58	1.01	2887.30
θ^M	−1.68	−2.42	−0.96	1.01	1918.89
τ	0.46	0.06	1.18	1.04	358.72
$\theta^M_{\text{School 1 (A)}}$	−0.13	−0.92	0.48	1.02	2385.22
$\theta^M_{\text{School 1 (B)}}$	0.07	−0.61	0.83	1.03	2741.46
$\theta^M_{\text{School 1 (C)}}$	0.14	−0.49	0.98	1.02	2442.56
$\theta^M_{\text{School 2 (D)}}$	−0.12	−0.95	0.52	1.03	2531.31
$\theta^M_{\text{School 2 (E)}}$	−0.06	−0.87	0.64	1.03	2720.45
θ^A	0.00	−1.88	1.87	1.01	3015.80
γ^{Absences}	−3.05	−7.56	0.55	1.01	2434.32
$\gamma^{\text{Community}R_t}$	0.51	−0.62	1.62	1.02	1316.72

ESS is the effective sample size, i. e. the number of independent MCMC samples with estimation power equivalent to the total number of autocorrelated samples[5], and $\hat{R}$ is the Gelman-Rubin convergence diagnostic[6]. Low ESS or $\hat{R}$ or $\hat{R} > 1.10$ indicate bad convergence of the model[7].

G.2 Estimated cumulative and new number of infections

Fig. Fa shows the estimated number of new infections over time in each class. The number of new infections increase visibly after mask mandates are lifted. An effect of air cleaners is not visually detectable, rather the number of new infections decrease towards zero as the number of susceptibles decrease (Fig. Fb).

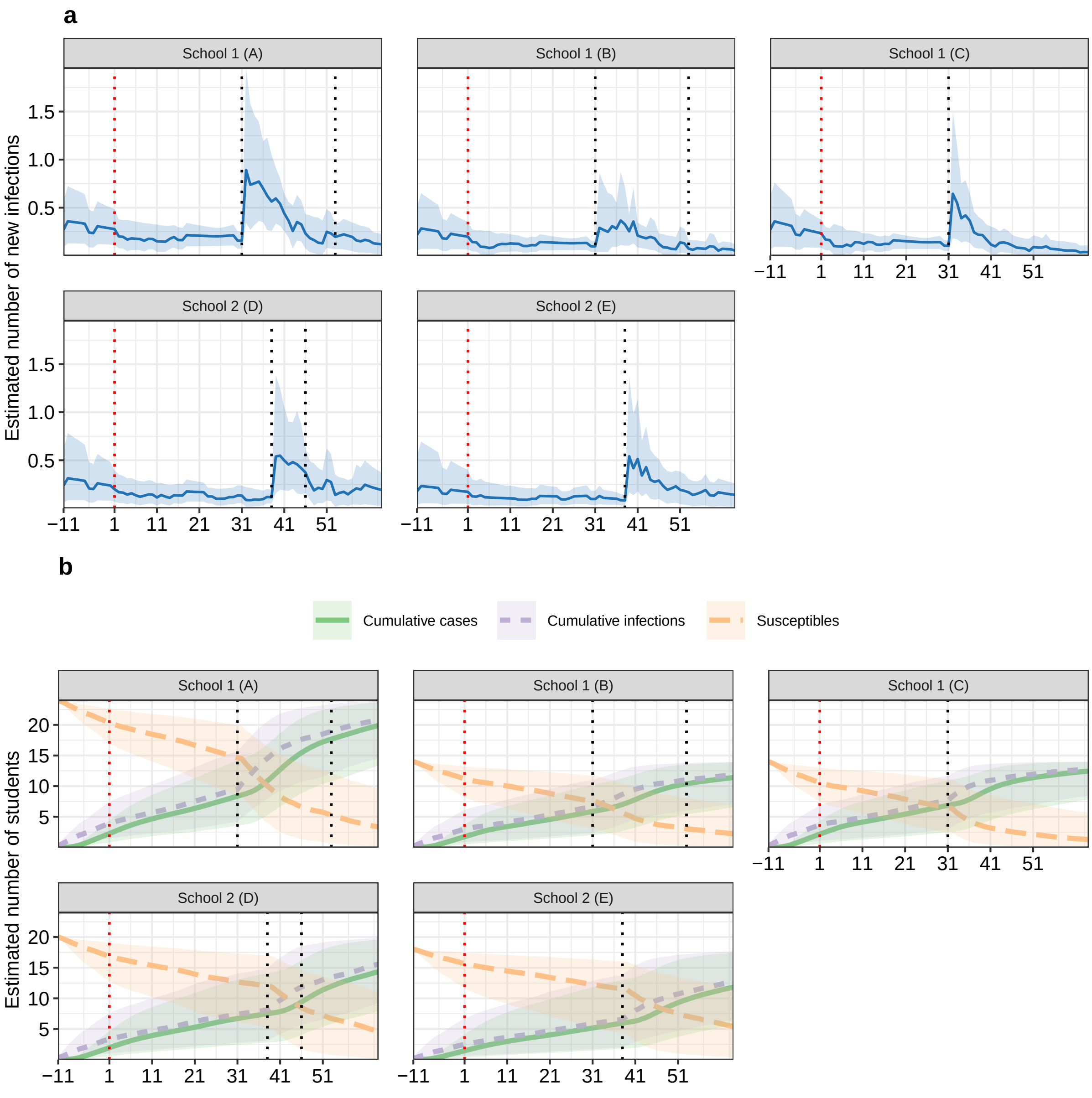

Fig F. Estimated incidence over time (posterior mean as line and 95%-CrI as shaded area). Dotted lines mark the start of the study (red) and intervention phases (black). **(a)** Number of new infections. **(b)** Cumulative number of cases and infections and number of susceptibles.

G.3 Overall model fit

Our model accounts for the delay from infection to case confirmation and allows transmission to change only at the dates of interventions. It thus reflects overall trends in transmission during study conditions rather than day-to-day variation. To evaluate how well our model fits these trends, Fig. G compares the estimated number of cases from the probabilistic simulation with the estimated number of new cases from the transmission model. Overall the estimates are in good agreement. The 95%-CrI of the model-based estimates includes the 95%-quantile of the simulation-based estimates.

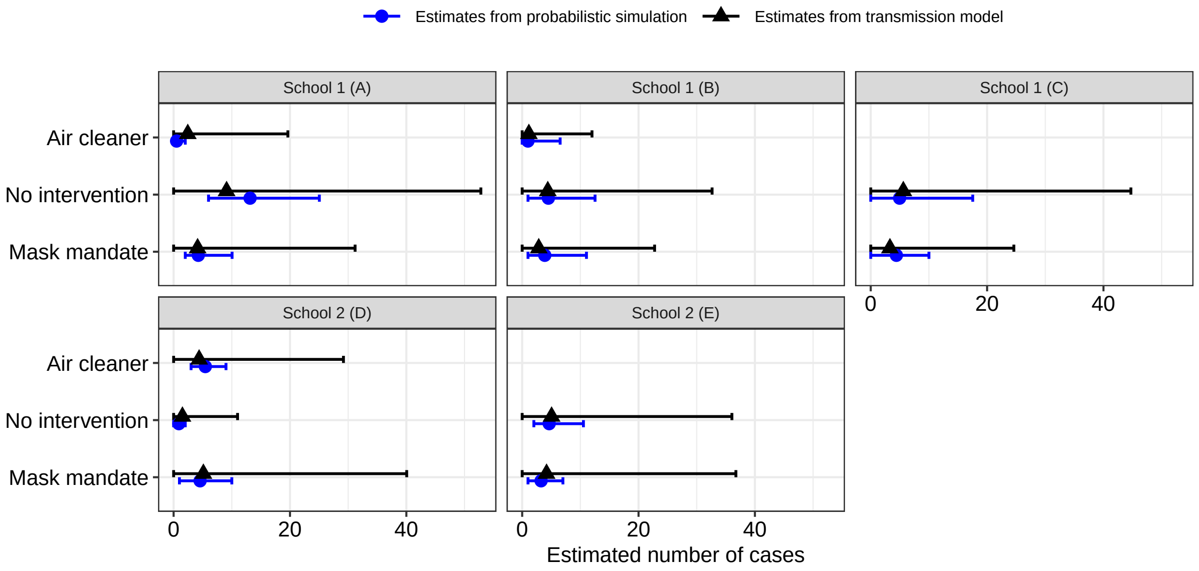

Fig G. Estimated (posterior mean as dot and 95%-CrI as line) number of cases from our probabilistic simulation (blue) and transmission model (black).

G.4 Estimated avoided infections for each generated dataset

In the main paper, we showed the estimated number of avoided infections summarized across all our 100 generated datasets. In Fig. H, we show the estimate for each individual dataset.

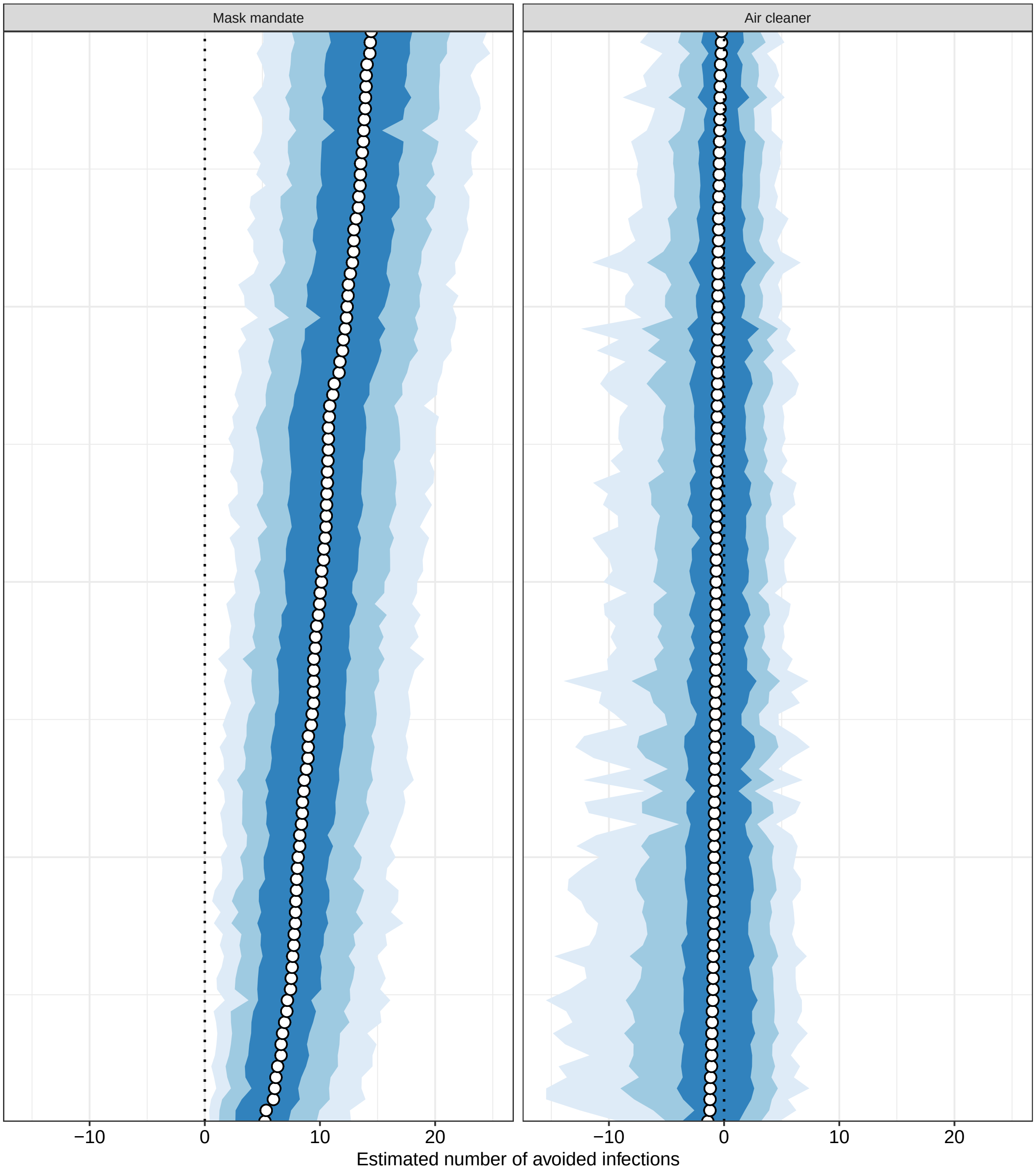

Fig H. Estimated number of avoided infections with interventions across classes (posterior mean as dot and 50%, 80%, and 95%-CrI as shaded areas) for each of the 100 generated datasets. Estimates shown in descending order of the magnitude of the mean effect.

Text H. Estimating changes in particle concentrations

The change in aerosol number concentration CN_{jt} in class j at day t (analogously for particle mass concentrations PM_{jt}) is estimated using Bayesian log-linear regression models, i. e.

$$\log CN_{jt} = \alpha + \beta\,\mathrm{School}_j + \boldsymbol{\gamma}\,\mathrm{Weekday}_t + \theta_M\,M_{jt} + \theta_A\,A_{jt} + \zeta\,\log \mathrm{N}_{jt} + \omega\,\log \mathrm{AER}_{jt}\ , \tag{14}$$

where α is the log of the aerosol concentration without interventions, β and $\boldsymbol{\gamma}$ are school and weekday effects, respectively, θ_M and θ_A are the effects of mask mandates (M_{jt}) and air cleaners (A_{jt}), respectively, ζ adjusts for the number of students in class (N_{jt}), and ω adjusts for the outdoor air exchange rate (AER_{jt}). The latter is computed from measured indoor CO_2 levels (see Section I). The percent reduction in particle concentrations with interventions are quantified as $100 \times (\mathrm{e}^{-\theta} - 1)\,\%$.

Text I. Computing rebreathed air volume and ventilation rat[illegible]

The rebreathed air volume (RAV), the ventilation (Q) and air exchange rate (AER) are computed from the measured CO_2 levels by making assumptions about respiratory volume per minute and the CO_2 concentration in the outdoor and exhaled air.

For RAV, we first compute the rebreathed air fraction[8] f as

$$f = (C - C_o)/C_a, \tag{15}$$

where C is the measured CO_2 concentration in the indoor air per minute (ppm) and C_o and C_a are the concentrations in the outdoor and exhaled air, respectively. The rebreated air fraction of other people f_o is computed as

$$f_o = f \cdot (n-1)/n, \tag{16}$$

where n is the number of people present in the indoor space. Finally, RAV is computed as

$$RAV = r \cdot f_o, \tag{17}$$

where $r = 8\,\mathrm{L/min}$ is the respiratory volume per minute[9], $C_o = 400\,\mathrm{ppm}$ is approximated by monitored levels in 2021 at a nearby location[10], and $C_a = 39,000\,\mathrm{ppm}$ is taken from the literature[8,9]

For Q and AER, we first compute the hourly ventilation rate Q as

$$Q = \frac{3600 \cdot G}{1e^{-3} \cdot (C - C_o)}, \tag{18}$$

where $G = 0.004\,\mathrm{L/s}$ is the CO_2 generation rate of children and adolescents[11]. Based on that, we compute the outdoor air exchange rate as

$$AER = Q \cdot n / V, \tag{19}$$

where V is the volume of the ventilated space ($207\,\mathrm{m}^3$ in School 1 and $233\,\mathrm{m}^3$ in School 2, respectively.

Text J. Results for changes in environmental variables

In the main paper, we showed results for changes in particle concentrations by study condition and summarized them across schools. In Fig. I, we present additional results disaggregated by schools and including other environmental variables. Furthermore, numerical estimation results for the reduction in particle concentrations with interventions are shown in Table H

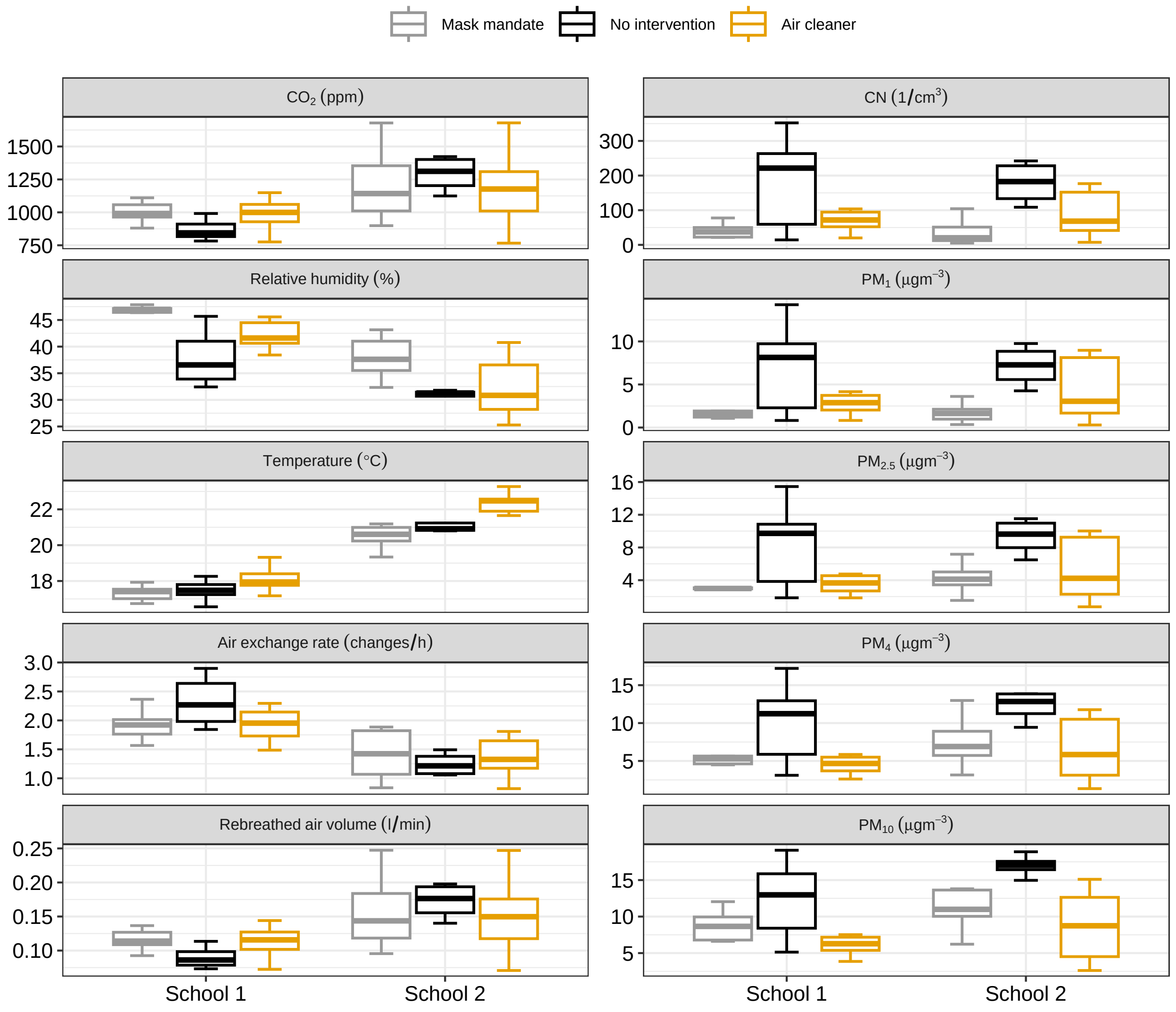

Fig I. Boxplot for the daily average values of each environmental variable by school and study condition.

Table H. Estimated reduction in aerosol number (CN) and particle mass (PM) concentrations with interventions (posterior mean and upper and lower estimate from the 95%-CrI).

	Mask mandate			Air cleaner		
Variable	Mean	Lower	Upper	Mean	Lower	Upper
CN	69.48	42.39	85.74	39.24	3.88	68.80
PM_1	65.77	40.08	82.24	41.05	6.62	67.98
$PM_{2.5}$	46.66	19.09	66.36	45.12	19.66	63.16
PM_4	33.82	7.83	54.49	46.49	27.26	61.72
PM_{10}	22.63	2.18	42.65	46.60	29.99	59.49

Text K. Modeling transmission risk of SARS-CoV-2 using a r

We model the risk of SARS-CoV-2 transmission in schools using the Wells-Riley[12] equation as modified by Rudnick and Milton[8]. According to this, transmission risk is measured as the probability of infection P, which can be estimated as

$$P = 1 - \exp\left(-\frac{f\,I\,q\,t}{n}\right), \tag{20}$$

where f is the rebreathed air fraction, I is the number of infectious individuals in space, n is the total number of individuals in space, q is the rate of generation of infectious quanta, and t is the duration of exposure.

We want to compare transmission risk between study conditions. For this, we assume that mask mandates and air cleaners reduce the emitted infectious quanta q. The relative reductions are quantified based on the estimated reductions in aerosol concentration (see Section J), i. e. a 69% (95%-CrI 42% to 86%) reduction with mask mandates and a 39% (95%-CrI 4% to 69%) with air cleaners. For comparison, McCreesh et al.[13] assumed a reduction of 75% (95%-CrI 56% to 85%) for masks.

Other input parameters are kept fixed. They are quantified based on our epidemiological and environmental data as follows:

- **f**: Following Rudnick and Milton[8], the rebreathed air fraction is computed from CO_2 levels as described in Section I. We use the average rebreathed air fraction $\bar{f}$ computed over the entire study period, which was 1.7%.

- **I**: The number of infectious students I at day t in class j are back-calculated from the observed number of cases in the following days. Let t_o be the day of symptom onset and t_a be the day of absence from school for case y. Further, let $p_\mathrm{I}(t - t_0)$ be the probability of being infectious at day t, which is computed as the relative probability of transmission based on the average infectiousness profile of SARS-CoV-2[14]. Then, I_{jt} is back-calculated as $I_{jt} = \sum_{y \in Y_j} \mathbb{I}_{t_y^a > t} \cdot p_\mathrm{I}(t - t_y^o)$, where $\mathbb{I}$ equals 1 if the case was recorded after t, otherwise 0. Of note, we compute the number of infectious students I_{jt} for each of our 100 generated datasets (see Section D). We then use the average across datasets for further analysis. Furthermore, we

exclude from the analysis the week before vacation and the week before the end of the study. The reason is that I_{jt} would be underestimated in these weeks as no information on cases of COVID-19 are collected during vacation and after the end of the study period.

- **n**: The number of people in the space n is the number of students present in the classroom plus one teacher.

- **I/n**: We use the average share of infectious people in space $\overline{I/n}$ computed over the entire study period, which was 4.5%.

We model the risk of infection assuming an $t = 6$ hour school day. We compare transmission risk for different activities, i. e. breathing (4.29×10^{-1} quanta/h), speaking (6.98×10^{-1} quanta/h), and singing (1.50×10^{0} quanta/h)[15]. These quantas are reduced in proportion to the reductions in aerosol concentration with mask mandates and air cleaners (see above). Based on that, the daily risk of infection was 1.0% (95%-CrI 0.4% to 1.9%) with mask mandates and 1.9% (95%-CrI 1.0% to 3.0%) with air cleaners, compared with a 3.1% risk without interventions (Fig. J).

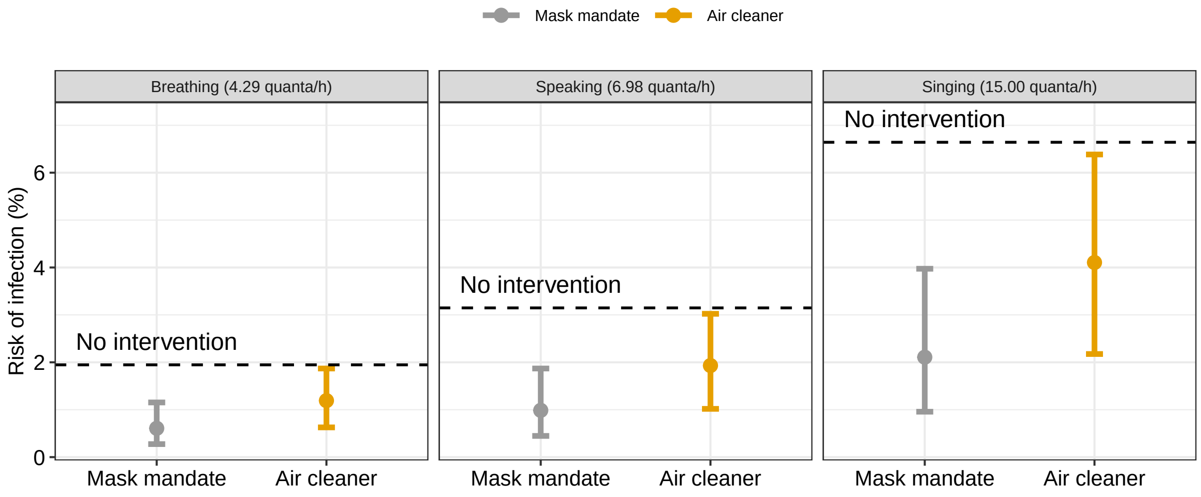

Fig J. Risk of infection (mean as dot, 95%-CrI as interval) using a modified Wells-Riley equation for a typical six hour school day by different activity levels corresponding to different emission rates of infectious quanta based on published estimates[15].

References

1. Scire J, Huisman JS, Angst D, Li J, Neher R, Maathuis M, et al.. Effective reproductive number (Re) in Switzerland; 2022. Available from: https://github.com/covid-19-Re/dailyRe-Data [last accessed 2022-09-30].

2. McAloon C, Collins A, Hunt K, Barber A, Byrne AW, Butler F, et al. Incubation period of COVID-19: A rapid systematic review and meta-analysis of observational research. BMJ Open. 2020;10(8):e039652. doi:10.1136/bmjopen-2020-039652.

3. Stan Development Team. Prior choice recommendations; 2020. Available from: https://github.com/stan-dev/stan/wiki/Prior-Choice-Recommendations [last accessed 2023-02-15].

4. Carpenter B, Gelman A, Hoffman MD, Lee D, Goodrich B, Betancourt M, et al. Stan: A probabilistic programming language. J Stat Softw. 2017;76:1–32. doi:10.18637/jss.v076.i01.

5. Stan Development Team. Stan modeling language users guide (Version 2.21); 2022. Available from: https://mc-stan.org/docs/2_21/reference-manual/effective-sample-size-section.html [last accessed 2023-02-15].

6. Gelman A, Rubin DB, others. Inference from iterative simulation using multiple sequences. Stat Sci. 1992;7(4):457–472. doi:10.1214/ss/1177011136.

7. Gelman A, Carlin JB, Stern HS, Dunson DB, Vehtari A, Rubin DB. Bayesian data analysis. Chapman & Hall; 2013.

8. Rudnick SN, Milton DK. Risk of indoor airborne infection transmission estimated from carbon dioxide concentration. Indoor Air. 2003;13(3):237–245. doi:10.1034/j.1600-0668.2003.00189.x.

9. Emmerich SJ, Persily AK. State-of-the-art review of CO2 demand controlled ventilation technology and application. DIANE; 2003.

10. National Oceanic & Atmospheric Administration. Earth System Research Laboratory; Global Monitoring Division; 2022. Available from: https://gml.noaa.gov/aftp/data/trace_gases/co2/flask/surface/txt/co2_hpb_surface-flask_1_ccgg_month.txt [last accessed 2022-10-06].

11. Persily A, de Jonge L. Carbon dioxide generation rates for building occupants. Indoor Air. 2017;27(5):868–879. doi:10.1111/ina.12383.

12. Riley RL, Mills CC, O'Grady F, Sultan LU, Wittstadt F, Shivpuri DN. Infectiousness of air from a tuberculosis ward. Am Rev Respir Dis. 1962;85(4):511–525. doi:10.1164/arrd.1962.85.4.511.

13. McCreesh N, Karat AS, Baisley K, Diaconu K, Bozzani F, Govender I, et al. Modelling the effect of infection prevention and control measures on rate of Mycobacterium tuberculosis transmission to clinic attendees in primary health clinics in South Africa. BMJ Global Health. 2021;6(10):e007124. doi:10.1136/bmjgh-2021-007124.

14. He X, Lau EHY, Wu P, Deng X, Wang J, Hao X, et al. Temporal dynamics in viral shedding and transmissibility of COVID-19. Nat Med. 2020;26(5):672–675. doi:10.1038/s41591-020-0869-5.

15. Buonanno G, Morawska L, Stabile L. Quantitative assessment of the risk of airborne transmission of SARS-CoV-2 infection: Prospective and retrospective applications. Environ Int. 2020;145:106112. doi:10.1016/j.envint.2020.106112.
